# Preimaginal development of *Aedes aegypti* L. (Diptera: Culicidae) in brackish water gives rise to adult mosquitoes with thicker cuticles and greater insecticide resistance

**DOI:** 10.1111/mve.12799

**Published:** 2025-03-07

**Authors:** Kokila Sivabalakrishnan, Andrew Hemphill, S. H. P. Parakrama Karunaratne, Arunasalam Naguleswaran, Isabel Roditi, Sinnathamby N. Surendran, Ranjan Ramasamy

**Affiliations:** ^1^ Department of Zoology, Faculty of Science University of Jaffna Jaffna Sri Lanka; ^2^ Institute of Parasitology, Vetsuisse Faculty University of Bern Bern Switzerland; ^3^ Department of Zoology, Faculty of Science University of Peradeniya Peradeniya Sri Lanka; ^4^ Institute of Animal Pathology, Vetsuisse Faculty University of Bern Bern Switzerland; ^5^ Institute of Cell Biology, Faculty of Science University of Bern Bern Switzerland; ^6^ IDFISH Technology Milpitas California USA

**Keywords:** *Aedes aegypti* L, arboviral diseases in coastal zones, brackish water mosquito habitats, coastal environmental changes affecting mosquito‐borne diseases, dengue and other arboviral diseases, insecticide resistance, mosquito cuticle structure, mosquito vector control, pyrethroid, organophosphate, and carbamate insecticides, salinity tolerance in mosquitoes

## Abstract

*Aedes aegypti* L. and *Aedes albopictus* Skuse mosquitoes (Diptera: Culicidae), the principal vectors of many human arboviral diseases, lay eggs and undergo preimaginal development in fresh water. They have recently been shown to also develop in brackish water in coastal areas. Previous findings showed that *Ae. aegypti* larvae developing in brackish water possessed thicker cuticles and greater larvicide resistance than larvae developing in fresh water. The present study compared cuticle ultrastructure, resistance to adulticides, and the activities of adulticide detoxifying enzymes in female mosquitoes emerging from fresh and brackish water‐developing *Ae. aegypti* preimaginal stages. The results showed that brackish water‐derived females possessed significantly thicker tarsal and abdominal cuticles compared to fresh water‐derived females. Brackish water‐derived *Ae. aegypti* females were also significantly more resistant to three different types of pyrethroids and malathion compared to fresh water‐derived females. Corresponding reversal of cuticle changes and adulticide resistance when preimaginal salinity was reversed showed that preimaginal salinity determined both procuticle structure and adulticide resistance in brackish water‐derived females. Compared with fresh water‐derived *Ae. aegypti* females, brackish water‐derived females had similar activities of the adulticide‐detoxifying enzyme families of esterases and glutathione S‐transferases and a modest increase in the activity of monooxygenases, all of which were lower than the threshold values attributed to resistance in field populations of *Ae. aegypti*. Reduced permeability of the thicker and remodelled cuticles in brackish water‐derived *Ae. aegypti* females to adulticides is proposed to be mainly responsible for their greater resistance to different types of adulticides. Greater salinity tolerance of preimaginal stages, adult cuticle changes and higher larvicide and adulticide resistance are inherited properties of brackish water‐developing *Ae. aegypti* that reverse in a few generations after transfer to fresh water. This is compatible with a role for epigenetic changes in the adaptation of *Ae. aegypti* to brackish water. Greater resistance of salinity‐tolerant *Ae. aegypti* to adulticides and larvicides poses a hitherto unappreciated problem for controlling arboviral diseases, with attendant implications also for other mosquito‐borne diseases.

## Graphical abstract

- The principal arboviral vector *Aedes aegypti* can develop in coastal brackish water field habitats (0.5–15 g/L salt) with larvae possessing thicker cuticles and greater resistance to the larvicide Temephos.
- Females emerging from brackish water‐developing preimaginal stages are now shown to have thicker and remodelled leg and abdominal cuticles and greater resistance to adulticides.
- This advance in mosquito vector biology highlights an important need for extending vector control measures to coastal brackish‐water habitats.

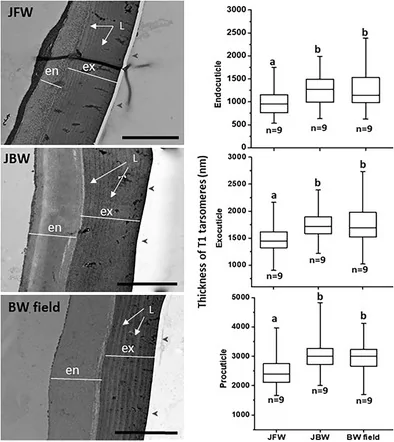

## INTRODUCTION

*Aedes aegypti* L. (Diptera: Culicidae), the principal global mosquito vector of arboviral diseases such as dengue, chikungunya, yellow fever and Zika, is widely held to oviposit and undergo preimaginal development only in fresh water (FW) (US CDC, 2023; WHO, 2009a & 2024). Applying larvicides and minimizing the preimaginal FW habitats of *Ae. aegypti* and the secondary arboviral vector *Aedes albopictus* Skuse, together with space spraying with adulticides during epidemics, are essential measures for controlling arboviral diseases (US CDC, 2023; WHO, 2009a, 2009b, 2024). Dengue is the most prevalent arboviral disease in the world. The World Health Organization (WHO) reported half the global population to be at risk of infection and 6.5 million cases resulting in 7300 deaths worldwide from dengue in 2023 (WHO, 2024). Dengue‐endemic Sri Lanka and its northern peninsular Jaffna district (Figure 1) recorded 105,049 and 8261 dengue cases, respectively, in 2019, with sharp decreases in 2020 and 2021 during the COVID‐19‐related restrictions on the movement of people, followed by a gradual return to pre‐pandemic levels in 2022 (Surendran, Nagulan, Sivabalakrishnan, et al., 2022; Surendran, Nagulan, Tharsan, et al., 2022).

**FIGURE 1:**
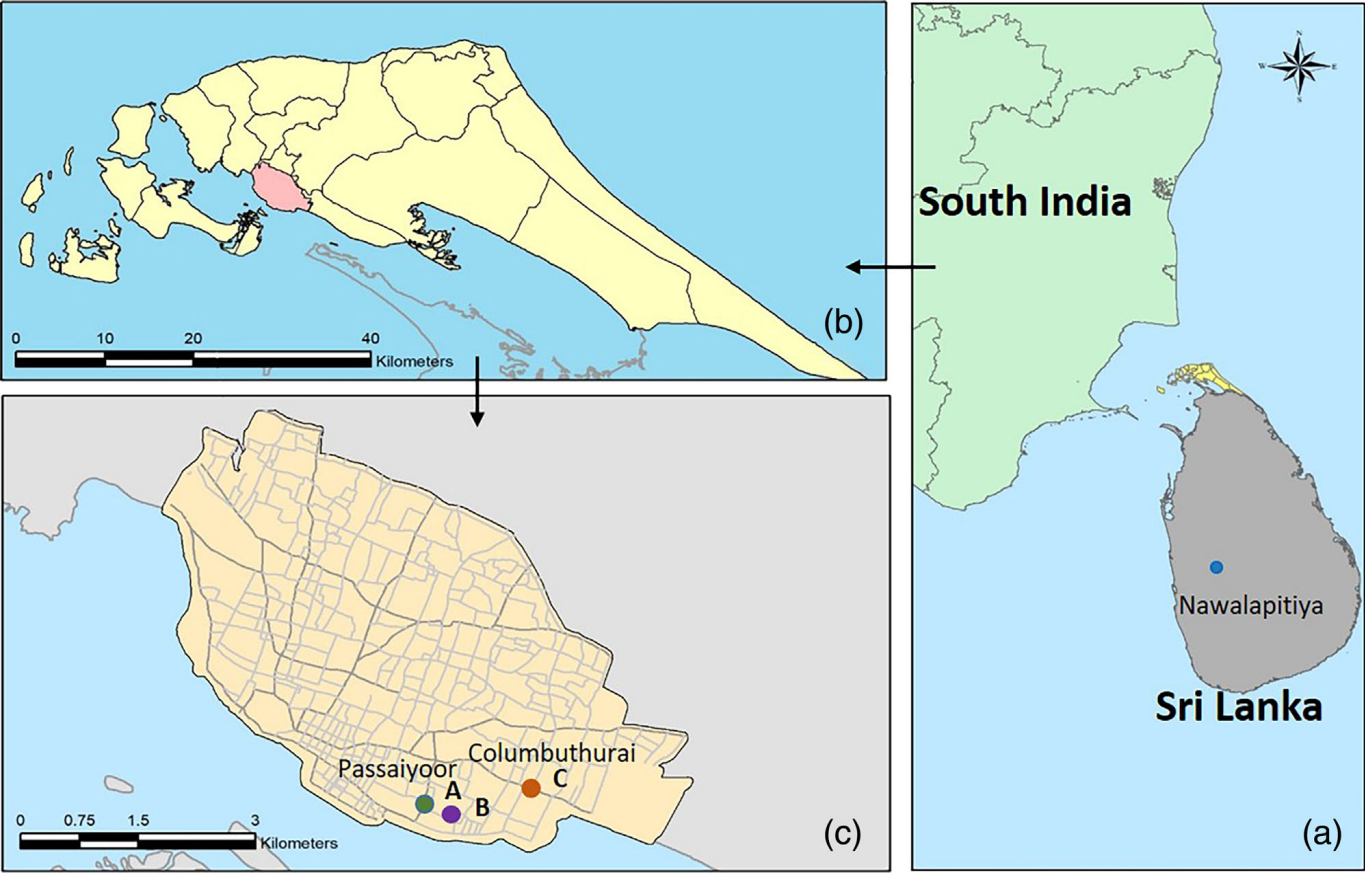
Map of study location and *Aedes aegypti* collection sites. (a) Location of the island of Sri Lanka and its Jaffna peninsula (highlighted in yellow) in relation to South India. Also shown is Nawalapitiya town in the central hills of Sri Lanka where *Ae. aegypti* larvae were collected to establish the fresh water laboratory colony termed NFW; (b) Jaffna peninsula with Jaffna city highlighted in beige; (c) Jaffna city showing the sites Passaiyoor A (9°38′53.6″N 80°01′49.8″ E) and B (9°38′05.4″N 80°01′56.1″ E), and Columbuthurai C (9°39′08.5″N 80°02′34.4″ E) where *Ae. aegypti* larvae were collected from brackish water field habitats.

*Aedes aegypti* and *Ae. albopictus* in the Jaffna peninsula oviposit and undergo preimaginal development not only in FW, but also in coastal brackish water (BW) collections of up to 15 g/L salt, for example, in fishing boats, beach debris and coastal wells (Jude et al., 2012; Ramasamy et al., 2011; Sivabalakrishnan et al., 2023; Surendran et al., 2012; Surendran et al., 2021; Surendran, Jayadas, et al., 2019). Both *Aedes* vectors have also been shown to naturally develop in similar BW field habitats in coastal areas of other countries (Supplementary Information, Table S1). FW, BW and saline water are respectively defined as containing <0.5, 0.5–30 and >30 g/L salt (Ramasamy et al., 2011, 2014). FW‐derived *Ae. aegypti* and *Ae. albopictus* larvae in the Jaffna peninsula are more salinity‐tolerant than the corresponding FW‐derived larvae from the mainland of Sri Lanka (Ramasamy et al., 2011). This has been attributed to the absence of a barrier to gene flow within the small (1100 km^2^) Jaffna peninsula and the peninsula's relative geographical isolation (Ramasamy et al., 2011; Sivabalakrishnan et al., 2023).

A cuticle covers the external surface of mosquitoes, the gut lumen excluding the midgut and the tracheal lumen (Bradley, 1987; Moussian, 2010; Ramasamy et al., 2021; Richards, 1978; Sivabalakrishnan et al., 2023). Among other functions, the external cuticle provides surface protection, senses the environment, supports muscle attachment, prevents desiccation in adults and acts as a barrier for ion and water exchange between preimaginal stages and their aqueous environment (Moussian, 2010; Richards, 1978; Willis, 2010). Mosquito cuticles are composed of (i) a thin chitin‐free epicuticle, not readily visible by transmission electron microscopy (TEM), and comprised of highly cross‐linked proteins overlaid with a lipid envelope rich in long chain hydrocarbons and waxy esters, and (ii) a wider, TEM‐visible inner procuticle containing chitin and proteins, and composed of an exocuticle and an endocuticle (including its more electron lucent layer sometimes termed mesocuticle) typically synthesized before and after ecdysis respectively (Moussian, 2010; Richards, 1978; Willis, 2010). Altered cuticle protein composition and increased transcription of genes concerned with the synthesis of specific cuticle components were prominent differences in fourth instar (L4) larvae of BW‐adapted *Ae. aegypti* compared with FW *Ae. aegypti* (Ramasamy et al., 2021). Adaptation of *Ae. aegypti* larvae to salinity is also accompanied by other changes, details of which are provided in Supplementary Information (Table S2). Importantly, BW *Ae. aegypti* L3 and L4 exhibited reduced susceptibility to temephos (Sivabalakrishnan et al., 2023). Temephos is the principal larvicide used worldwide for controlling *Aedes* vectors (US CDC, 2023; WHO, 2009a, 2009b, 2024).

Adaptation of *Ae. aegypti* and *Ae. albopictus*, and others regarded as typical FW mosquitoes such as the malaria vectors *Anopheles culicifacies* and *Anopheles stephensi*, to develop in BW (Jude et al., 2010; Jude et al., 2012; Surendran, Sivabalakrishnan, et al., 2019), heightens the risk of mosquito‐borne disease transmission in coastal areas because BW habitats are globally neglected in mosquito vector control programmes (Ramasamy, 2024). Greater disease transmission in coastal areas can readily spread to adjacent inland areas (Ramasamy & Surendran, 2011, 2012, 2016). Increasing salinization of groundwater in coastal zones due to rising sea levels, accumulation of anthropogenic container debris in littoral areas and the unsustainable extraction of groundwater can expand the availability of coastal BW habitats for mosquito vectors (Ramasamy & Surendran, 2011 & 2012). This is already evident in the Jaffna peninsula (Surendran et al., 2020). The expansion of BW habitats of mosquito vectors is of worldwide concern because about 10% of the global population lives in coastal areas (Ramasamy & Surendran, 2011 & 2012).

A few mosquito species that are vectors of human disease and found in coastal locations normally oviposit and undergo preimaginal development in BW, but details of their cuticle structure and susceptibility to adulticides are not known (Bradley, 1987; Ramasamy & Surendran, 2011 & 2012). We therefore investigated whether cuticle ultrastructure and adulticide susceptibility are altered in adult females emerging from preimaginal stages of *Ae. aegypti* developing in BW compared to FW.

## MATERIALS AND METHODS

### *Aedes aegypti* laboratory colonies

Self‐mating Jaffna brackish water (JBW) and Jaffna fresh water (JFW) laboratory colonies of *Ae. aegypti* were initiated with larvae collected from multiple BW and FW habitats in the Jaffna peninsula (Figure 1) as previously described (Ramasamy et al., 2011 & 2014; Sivabalakrishnan et al., 2023). Preimaginal stages of JBW and JFW *Ae. aegypti* were respectively maintained in seawater diluted with tap water to yield 10 g/L salt and potable tap water (0 g/L salt) obtained from an artesian well in the centre of the peninsula. Because FW *Ae. aegypti* in the Jaffna peninsula were more salinity‐tolerant than FW mosquitoes from the mainland of Sri Lanka, and JFW and JBW mosquitoes were reproductively compatible (Ramasamy et al., 2011; Sivabalakrishnan et al., 2023), we also initiated a laboratory colony termed Nawalapitiya fresh water (NFW) with *Ae. aegypti* larvae collected from two different sites in Nawalapitiya in the central hills of mainland Sri Lanka (220 miles from Jaffna, Figure 1), where mosquitoes are not exposed to BW habitats. The NFW colony was maintained in tap water purified by reverse osmosis in the insectary of the Department of Zoology, University of Jaffna. Larvae in colonies were reared in 30 × 25 cm plastic trays containing 1.5 litres of water with up to 150 larvae per tray and fed with fish meal powder twice a day. Adults and larvae were maintained at 28–30°C at a relative humidity of approximately 75% and 12 h dark and light periods. Adult mosquitoes were allowed to feed every 3 days on Balb/c mice and on 10% glucose pledgets at other times. Appropriate 10 g/L salt BW or 0 g/L salt FW surfaces for oviposition were provided in the cages after blood‐feeding, and eggs were harvested 2 days later as described (Ramasamy et al., 2011; Sivabalakrishnan et al., 2023). Stored eggs from early generations of colonies were used to renew the colonies as needed. The 66th generation (G66) of the JBW laboratory colony had a significantly higher 50% lethal concentration (LC_50_) of 16.0 g/L salt than the G66 JFW laboratory colony (LC_50_ of 11.6 g/L) for the L1 to adult transition (Sivabalakrishnan et al., 2023). Reciprocal crosses between G74 and G75 of JBW and JFW mosquitoes showed them to be reproductively compatible (Sivabalakrishnan et al., 2023). G17 of the NFW colony had an LC_50_ (11.2 g/L) for the L1 to adult transition, which was not significantly different from the JFW colony but significantly lower than the JBW colony (Sivabalakrishnan et al., 2023). Although morphological differences between the JBW and JFW adult females were not detectable on visual examination, the fresh weight of 20 sugar‐fed, 3‐day‐old females from the G84 JFW and JBW colonies was measured individually. The left wing of each mosquito was then dissected and placed on a glass slide. Wing length was determined from the axillary incision to the apical margin, using a CX21 (Olympus, Tokyo) light microscope equipped with an ocular micrometre, as a surrogate measure of mosquito size as previously described (Nasci, 1986).

To determine the effects of reversing the larval rearing salinity on adulticide susceptibility and cuticle structure, reversal colonies were formed from JBW and JFW colonies as described (Sivabalakrishnan et al., 2023). A reversal colony JBWR was established from the JBW colony by collecting approximately 600 eggs laid by 20–25 G63 JBW females on 0 g/L FW egg‐laying surfaces and then hatching and rearing larvae in FW. Similarly, a reversal colony JFWR was established from the G63 JFW colony by collecting approximately 600 eggs laid by 20–25 JFW females on 10 g/L salt BW egg‐laying surfaces and subsequently hatching and rearing larvae in 10 g/L salt BW. The proportions of eggs hatching into L1 and then maturing through L2, L3, L4 and pupa into adults were also determined for the first seven generations in the JBWR and JFWR colonies to assess the impact of reversing salinity.

### *Aedes aegypti* from brackish water field habitats in coastal areas of Jaffna city

*Aedes aegypti* larvae were collected during November and December 2023 from BW habitats in coastal areas of Jaffna city from two different discarded plastic containers with 6 g/L salt water at two sites in Passayoor (9°38′53.6″N 80°1′49.8″ E; 9°38′5.4″N 80°1′56.1″ E) and a discarded plastic yoghurt container with 8 g/L salt water in Columbuthurai (9°39′8.5″N 80°2′34.4″ E) (Figure 1). The salinity of larval habitats was measured with a hand‐held refracto‐salinometer (Atago, Tokyo, Japan). Larvae were collected with 5 mL plastic pipettes and brought to the insectary of the Department of Zoology, University of Jaffna, where they were morphologically identified as *Ae. aegypti* using published keys (Rueda, 2004) and reared to adulthood in the same water from the habitats where they were collected.

### Transmission electron microscopy to measure cuticle thicknesses

Three‐day‐old sugar‐fed BW female adult *Ae. aegypti* emerging from larvae collected from BW field habitats, and the G75 JBW, G75 JFW, G10 JBWR and G10 JFWR laboratory colonies, were processed for transmission electron microscopy (TEM).

Tarsomere 1 (T1) of the left middle leg and the 6th abdominal segment were dissected from non‐blood‐fed females, washed in 0.1 M Na‐cacodylate pH 7.3 buffer (Sigma‐Aldrich, MO, USA), and then immersed in cacodylate buffer containing 2% glutaraldehyde (Sigma‐Aldrich, MO, USA) for 12–15 h at 4°C. After three washes in cacodylate buffer, post‐fixation was performed overnight in 2% osmium tetroxide in cacodylate buffer pH 7.3. Specimens were then transferred to cacodylate buffer pH 7.3 and transported for further processing for TEM at the University of Bern, Switzerland. After three washes in water, specimens were dehydrated sequentially through a graded series of ethanol (30%, 50%, 70%, 90% and 3 × 100%, 2–5 min each). Following dehydration, samples were embedded in Epon812 epoxy resin using plastic embedding moulds (Sigma‐Aldrich, MO, USA). After polymerization of the resin at 60°C overnight, ultrathin (80 nm) sections were cut using an ultramicrotome (Reichert and Jung, Vienna, Austria). Sections were transferred onto formvar‐carbon‐coated 200 mesh nickel grids (Plano GmbH, Marburg, Germany), stained with Uranyless® and lead citrate (both from Electron Microscopy Sciences, Hatfield, PA, USA), and imaging was performed on a FEI Morgagni transmission electron microscope equipped with a 12‐megapixel Morada digital camera system operating at 80 kV.

T1 from nine females from each of the JBW and JFW colonies, three females from each of the three BW field habitats and three females from each of the JBWR and JFWR colonies were analysed by TEM. Abdomens from four females each from the JBW, JFW, JBWR and JFWR colonies, as well as two females from each of the three BW field habitats, were also analysed by TEM. T1 and abdomens for TEM were derived from a proportion of the same mosquitoes. Thin cross‐sections were examined in the mid‐region of T1 from the left middle leg and the middle of the 6th abdominal segment. Thicknesses of the endocuticle, exocuticle and procuticle from EM images were separately measured, referring to the scale bar provided with each image and applying ImageJ (Schindelin et al., 2012). Measurements were taken at 20 different sites distributed across each section of T1 and abdomen.

### Adulticide susceptibility of JBW, JFW, NFW, JBWR and JFWR of *Aedes aegypti* laboratory colonies

This was determined with adulticide concentrations and procedures recommended by the WHO for differentiating susceptible and resistant field populations of *Ae. aegypti* (WHO, 2016), as previously done in Sri Lanka (Karunaratne et al., 2013). Sugar‐fed, three‐ to five‐day old adult female mosquitoes of G63‐G72 JFW and JBW, G17‐G29 NFW and G7‐G13 JFWR and JBWR were tested against the recommended WHO discriminating dosage of deltamethrin (0.03%), malathion (0.8%), permethrin (0.25%), propoxur (0.1%) and Etofenprox (0.5%) (Karunaratne et al., 2013; WHO, 2016). The WHO bioassays use discriminating concentrations of each adulticide determined from a log‐probit mortality curve for a reference susceptible mosquito population and taking twice the concentration of adulticide that produced 100% mortality. The assays measure the resistance of mosquitoes to tarsal contact with adulticides dispersed in oil and sprayed on filter papers (Karunaratne et al., 2013; WHO, 2016). Mosquitoes in batches of 20 were allowed to rest on adulticide‐impregnated papers for 1 h at 28 ± 2°C and 75% relative humidity. The mosquitoes were then removed to different containers, and the numbers of dead mosquitoes were counted after a 24 h recovery period. Five replicates (20 mosquitoes per replicate) per adulticide were done, and experiments performed twice so that a total of 200 mosquitoes were tested with each adulticide. A total of 100 mosquitoes were similarly exposed to filter papers sprayed only with the corresponding solvent oil as a control for each adulticide (Karunaratne et al., 2013; WHO, 2016). Mortality in all control experiments was <5%.

### Assays for enzymes detoxifying adulticides in adult female *Aedes aegypti* from JBW and JFW laboratory colonies

Enzyme assays were performed according to standard WHO protocols (Karunaratne et al., 2013; Nugapola et al., 2020; WHO, 1998). Three‐day‐old sugar‐fed females from G77 JBW and JFW colonies were individually tested in duplicate for total protein and activities of non‐specific esterases (EST), glutathione S‐transferases (GST) and monooxygenases (MO). Each mosquito was homogenized in 200 μL ice‐cold distilled water; the homogenate was then centrifuged at 2000 *g* for 2 min, and the supernatant was used in assays (WHO, 1998). Absorbances were measured with a Bio‐Tek 800TS microplate reader (Bio‐Tek, Washington, USA).

Protein concentration in 10 μL of mosquito homogenate was measured with bovine serum albumin as the standard using a Bio‐Rad protein assay kit (Bio‐Rad, Hercules, USA) according to the manufacturer's protocol.

EST was assayed by mixing 10 μL of mosquito homogenate supernatant with 200 μL of 1 mM p‐nitrophenyl acetate solution in a microtiter plate well. The rate of reaction was followed at 12 s intervals at 405 nm for 2 min. EST activity was determined as micromole of p‐nitrophenol produced per minute per milligram of protein.

GST was assayed with 10 μL of mosquito homogenate supernatant mixed with 200 μL of freshly prepared reduced glutathione/1‐chloro‐2,4‐dinitrobenzene (CDNB) working solution in a microtiter plate well. The enzyme reaction rate was measured at 340 nm at 12 s intervals for 5 min. The GST specific activity per individual mosquito was determined as the micromole of CDNB‐conjugated glutathione formed per minute per milligram protein.

The MO activity was measured by adding 80 μL of 0.625 M potassium phosphate buffer (pH 7.2), 200 μL of a methanolic solution of 3,3,5,5‐tetramethyl benzidine solution and 25 μL of hydrogen peroxide (3% v/v) to 20 μL of centrifuged homogenate. Absorbance was read at 650 nm after a 2‐h incubation at ambient temperature. MO activity was measured as equivalent units of cytochrome calculated with a cytochrome c standard curve.

### Statistical analysis

The Kolmogorov–Smirnov normality test was conducted to assess the normal distribution of measurements using Minitab 17 software (Minitab LLC, State College, PA, USA).

Measured values for the thicknesses of the endocuticle, exocuticle and procuticle were not normally distributed for adults emerging from the larvae collected in BW field habitats. Therefore, the significance of differences in cuticle thicknesses between JFW, JBW and BW field mosquitoes was determined by the non‐parametric Mann–Whitney *U* test using Minitab 17 software with a significance threshold of *p* < 0.05 (Minitab LLC, State College, PA, USA).

Since the cuticle thickness measurements for each of the four laboratory colonies (JBW, JFW, JBWR, JFWR) were normally distributed, Tukey's method for multiple comparisons in the analysis of variance using Minitab 17 software (Minitab LLC, State College, PA, USA) was applied to compare the cuticle thicknesses between colonies, with a significance threshold of *p* < 0.05.

Tukey's method for multiple comparisons in the analysis of variance with Minitab 17 software (Minitab LLC, State College, PA, USA) was also used to compare the mean per cent mortality of the test populations to each adulticide, with the significance threshold set at *p* < 0.05. Susceptibility status was assigned as defined by the WHO for field mosquito vector populations: susceptible (≥ 98% mortality), possibly resistant (90%–97% mortality) and resistant (<90% mortality) (WHO, 2016).

Student's two‐sided t‐test was used to determine the significance of differences in the activities of adulticide detoxifying enzymes, wet weights and wing lengths between JBW and JFW females.

## RESULTS

### Cuticle ultrastructure in T1 tarsomeres of adult *Aedes aegypti* undergoing preimaginal development in brackish and fresh water

Cuticle ultrastructure was determined by TEM in cross‐sections of T1 from the middle‐left leg of adult female *Ae. aegypti* 3 days post‐eclosion. We examined and measured thicknesses in the exocuticle and endocuticle separately because they are considered to be largely synthesized before and after ecdysis respectively (Moussian, 2010; Richards, 1978). This was done on females from G75 JBW and G75 JFW laboratory colonies, and also on females emerging from larvae collected in the three different BW field habitats. Tarsi are the mosquito structures that make the greatest contact with adulticides when they rest on adulticide‐sprayed surfaces and treated bed nets (Karunaratne et al., 2013; WHO, 2016). Examples of T1 TEM images and the box plots of T1 cuticle thickness measurements from BW‐field, JBW and JFW mosquitoes are shown in Figure 2 with additional details in Supplementary Information (Table S3).

**FIGURE 2:**
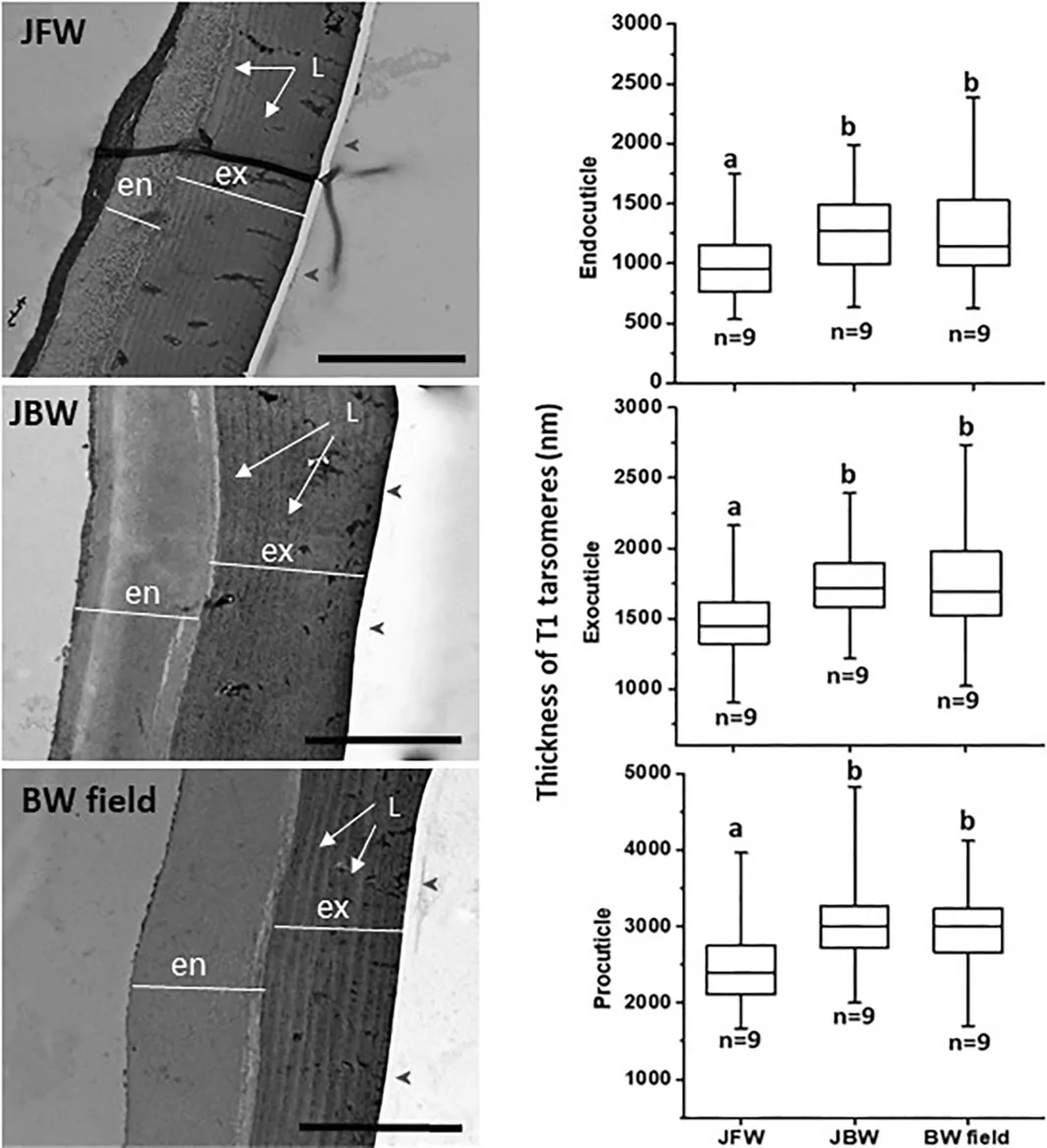
Comparison of endocuticle, exocuticle and procuticle in T1 tarsomeres of female *Aedes aegypti* emerging from JBW and JFW larvae and larvae collected from BW field habitats. en—endocuticle; ex—exocuticle; L—lamellae; n—number of different mosquitoes analysed with 20 measurements done on each mosquito; arrowheads indicate the external surface. Box plots show the range (whiskers), median (horizontal line) and 25th and 75th percentiles of measured thicknesses. Thickness measurements that do not share a letter within each cuticle structure were significantly different (*p* < 0.05) between the mosquito populations by the Mann–Whitney *U* test. Black scale bars represent 2000 nm.

The epicuticle was not visualizable in our TEM procedure. Because the endocuticle, exocuticle and procuticle thicknesses in the BW field‐derived *Ae. aegypti* females were not normally distributed, the significance of differences between the JBW, JFW and BW field populations was determined by the non‐parametric Mann–Whitney *U* test. The endocuticle, exocuticle and procuticle thicknesses in T1 were not significantly different from the corresponding structures in females derived from the JBW colony and BW field habitats, but these were significantly thicker than the corresponding structures in JFW colony females (Figure 2). For example, the median whole procuticle thicknesses in T1 of JBW and BW field females were 26% greater than in JFW females (Supplementary Table S3). Examination of the TEM images from multiple mosquitoes showed that electron‐dense lamellae, composed of chitin microfibrils and chitin‐binding cuticle proteins (Moussian, 2010), were more prominent in T1 exocuticles than in endocuticles. Consistent differences between JFW, JBW and BW field females in lamellae within either T1 exocuticles or endocuticles could not be identified.

We separately compared T1 cuticle ultrastructure in females from the two reversal colonies JBWR and JFWR with those of the parent JBW and JFW colonies (Figure 3 with additional details in Supplementary Table S3). Endocuticle, exocuticle and procuticle thicknesses in each of the four laboratory colonies were normally distributed and, therefore, the significance of differences in thicknesses between different colonies was determined by Tukey's method for multiple comparisons in the analysis of variance.

**FIGURE 3:**
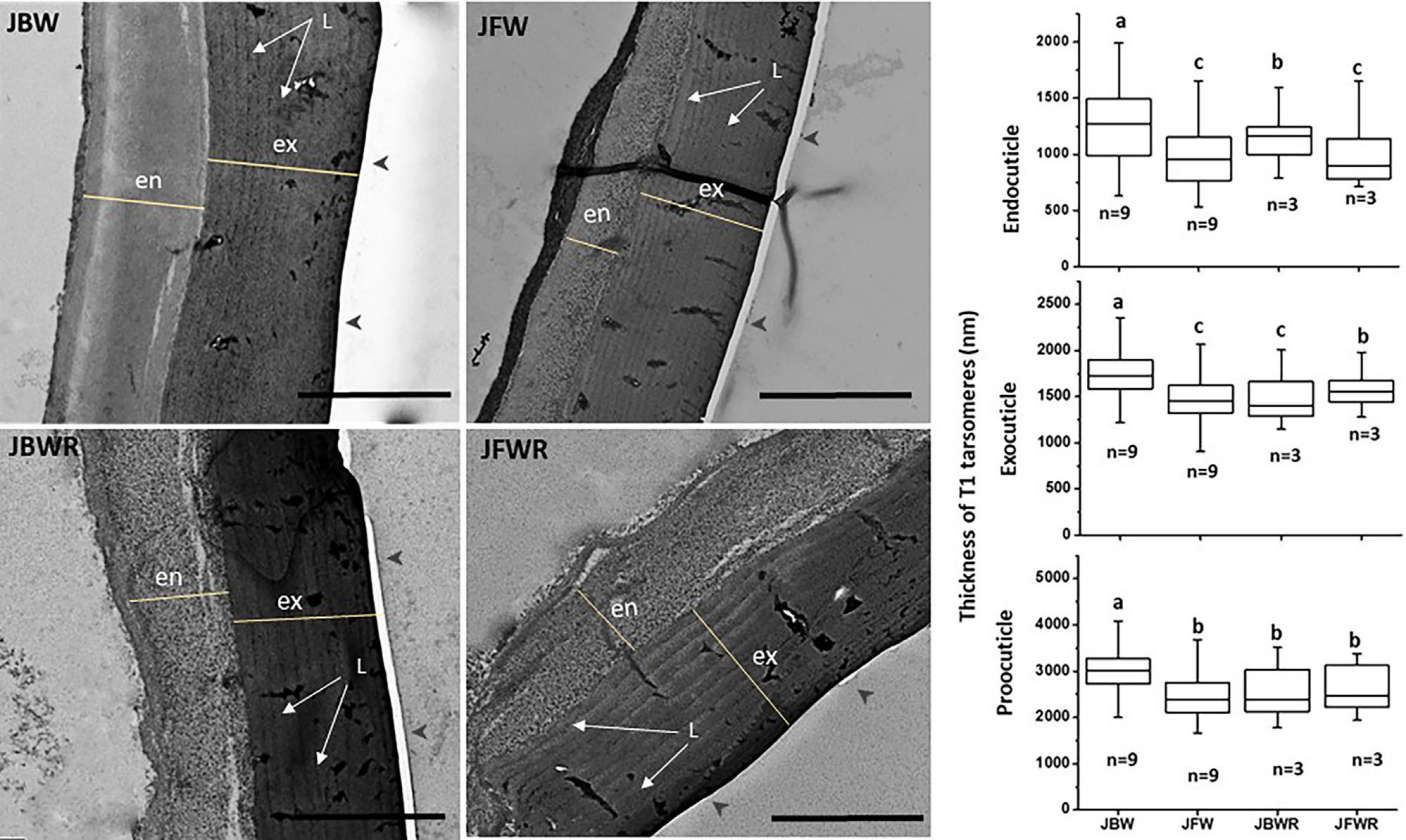
Comparison of endocuticle, exocuticle and procuticle in T1 tarsomeres of female *Aedes aegypti* emerging from JBW, JFW JBWR and JFWR larvae. en—endocuticle; ex—exocuticle; L—lamellae; n—number of different mosquitoes analysed with 20 measurements done on each mosquito; arrowheads indicate the external surface. Box plots show the range (whiskers), median (horizontal line) and 25th and 75th percentiles of measured thicknesses. Values that do not share a letter within each cuticle structure were significantly different (*p* < 0.05) between the mosquito populations by Tukey's test. Black scale bars represent 2000 nm.

After 10 generations of development in FW, the procuticle and exocuticle thicknesses of JBWR female T1s had reduced to the thicknesses of the corresponding structures in JFW females, while the JBWR female T1 endocuticle had an intermediate thickness between the T1 endocuticles of JFW and JBW females.

After 10 generations of development in BW, the T1 exocuticle of JFWR females was of an intermediate thickness and significantly different from the exocuticles in JBW and JFW females. The T1 procuticle and endocuticle thicknesses in JFWR females were not significantly different from the corresponding structures in JFW females but were significantly thinner than the corresponding structures in JBW females.

These findings show that when preimaginal rearing salinity is reversed for 10 generations, the procuticle, endocuticle and exocuticle in T1 tend to assume thicknesses more characteristic of the new FW environment in JBWR females, but that the corresponding changes in their new BW environment were less evident in JFWR females. Consistent differences in lamellar structure within either endocuticles or exocuticles were not observed when comparing T1 TEM images from multiple JBW, JFW, JBWR and JFWR females.

### Cuticle ultrastructure in abdomens of female *Aedes aegypti* undergoing preimaginal development in brackish and fresh water

The abdomen potentially provides a large surface for absorbing adulticides applied as space sprays. TEM was therefore done on mid‐6th abdominal segments. Measured cuticle thicknesses and examples of TEM images of abdominal cross‐sections of JBW, JFW and BW field mosquitoes are shown in Figure 4 with additional details in Supplementary Information (Table S4).

**FIGURE 4:**
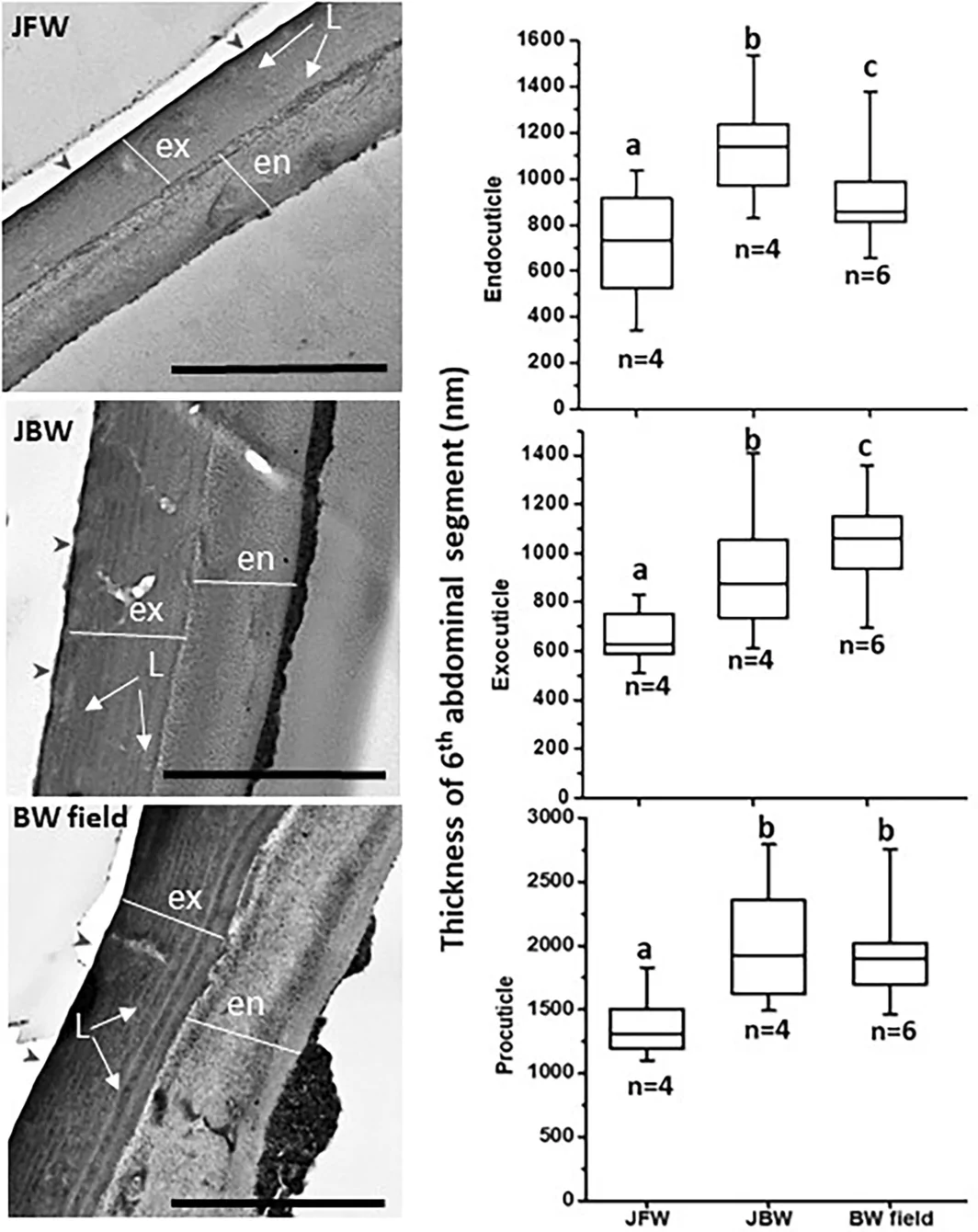
Comparison of endocuticle, exocuticle and procuticle in the 6th abdominal segments of female *Aedes aegypti* emerging from JBW and JFW larvae and larvae collected from BW field habitats. en—endocuticle; ex—exocuticle; L—lamellae; n—number of different mosquitoes analysed with 20 measurements in each mosquito; arrowheads indicate the external surface. Box plots show the range (whiskers), median (horizontal line) and 25th and 75th percentiles of measured thicknesses. Values that do not share a letter within each cuticle structure were significantly different (*p* < 0.05) between the mosquito populations by the Mann–Whitney *U* test. Black scale bars represent 2000 nm.

The JBW colony and BW field females possessed significantly thicker abdominal endocuticles, exocuticles and procuticles than the corresponding structures in JFW colony females. For example, median values showed that whole procuticles in JBW and BW field females were respectively 47% and 46% thicker than in JFW females (Supplementary Information Table S4). The abdominal procuticle thicknesses in the JBW colony and BW field *Ae. aegypti* were not significantly different. Abdominal cuticles were also thinner than T1 cuticles in the corresponding BW and FW female *Ae. aegypti* populations. TEM images of the abdomen showed more prominent lamellae in exocuticles than endocuticles as in T1. However, in contrast to T1, lamellae tended to be more prominent in the exocuticles of abdomens from JBW and BW field‐derived females than in JFW females on comparing multiple TEM images from the different mosquitoes.

We also compared abdominal cuticle ultrastructure in females from the two reversal colonies JBWR and JFWR with those of the parent colonies JBW and JFW (Figure 5, with additional details in Supplementary Information Table S4).

**FIGURE 5:**
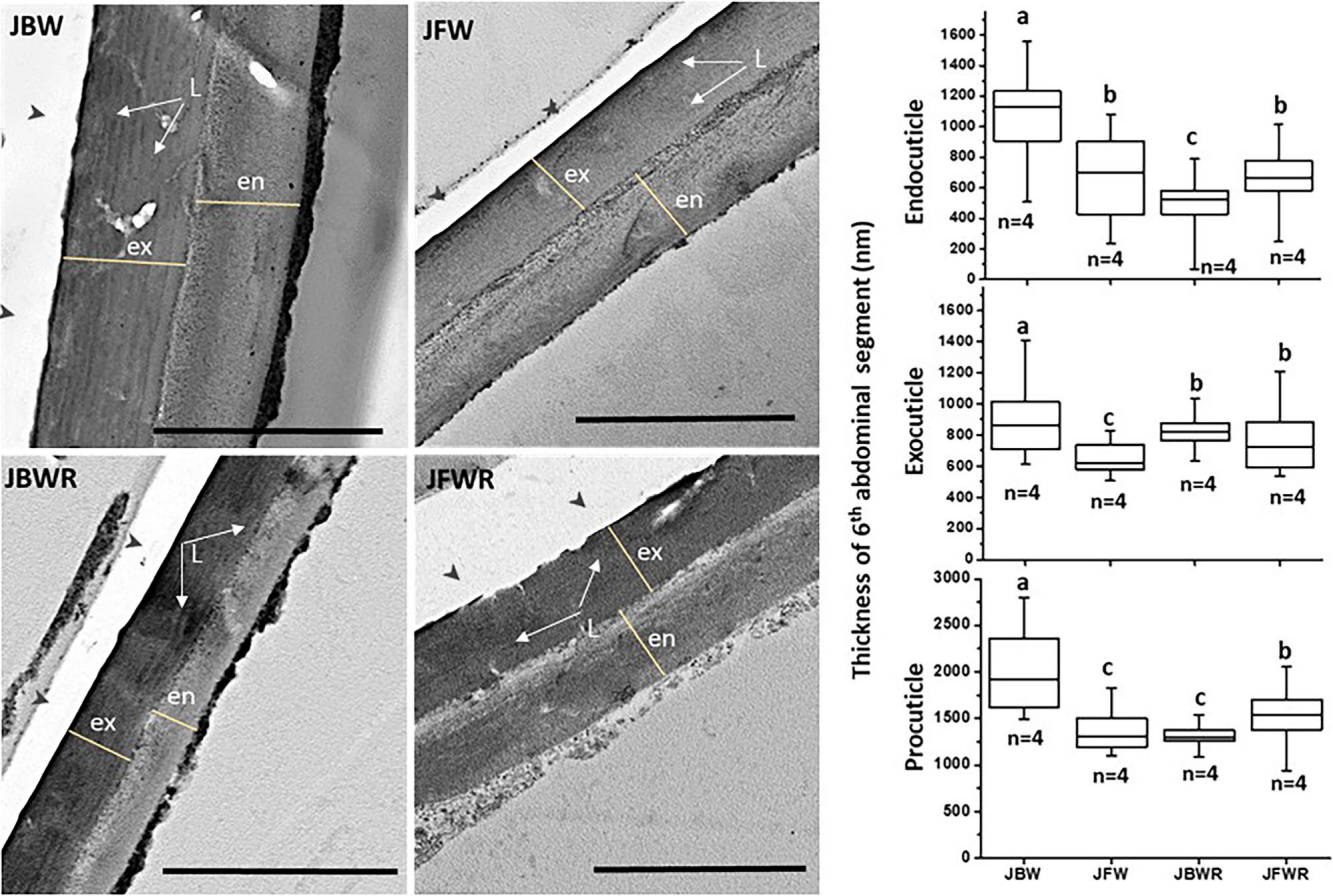
Comparison of endocuticle, exocuticle and procuticle ultrastructure in the 6th abdominal segments of female *Aedes aegypti* emerging from JBW, JFW JBWR and JFWR larvae. en—endocuticle; ex—exocuticle; L—lamellae; n—number of different mosquitoes analysed with 20 measurements in each mosquito; arrowheads indicate the external surface. Box plots show the range (whiskers), median (horizontal line) and 25th and 75th percentiles of measured thicknesses. Values that do not share a letter within each cuticle structure are significantly different (*p* < 0.05) between the mosquito populations by Tukey's test. Black scale bars represent 2000 nm.

After developing for 10 generations in FW, the procuticle thickness of JBWR female abdomens was not significantly different from JFW females and significantly thinner than in JBW females. The endocuticle and exocuticle of JBWR female abdomens were significantly thinner than the corresponding structures in JBW females and respectively thinner and thicker than the corresponding structures in JFW females.

After 10 generations of development in BW, the abdominal procuticle and exocuticle of JFWR females were of intermediate thickness between the corresponding structures in JBW and JFW females. However, the abdominal endocuticle thickness in JFWR was not significantly different from that in JFW females.

Consideration of multiple TEM images from different mosquitoes showed that exocuticle lamellae in abdomens tended to be more prominent in JBWR than in JFW females, and in JBW than in JFWR females, but these differences were less marked than those between JBW and JFW females.

Therefore, when preimaginal rearing salinity is reversed for 10 generations, the endocuticle and exocuticle as well as the whole procuticle of female abdomens appear to assume properties in their new environment that are characteristic of established JBW and JFW colonies. However, the changes were more marked in BW‐adapted mosquitoes transferred to FW (JBWR) than in FW‐adapted mosquitoes transferred to BW (JFWR).

### Insecticide susceptibilities of adult females from *Aedes aegypti* brackish and fresh water laboratory colonies and their reversal colonies

We assessed the susceptibilities to different classes of common adulticides of three‐ to five‐day‐old non‐blood‐fed females from the G63‐G72 JBW and JFW, G17‐G29 NFW, the G7‐G13 JBWR and JFWR colonies. Susceptibilities to permethrin (a type 1 pyrethroid lacking a α‐cyano group), etofenprox (a synthetic type 1 pyrethroid where an ester is replaced with an ether bond), deltamethrin (a type 2 pyrethroid containing a α‐cyano group), malathion (an organophosphate) and propoxur (a carbamate) were determined by conventional WHO bioassays for determining resistance in field populations of mosquito vectors. Table 1 shows the results of bioassays performed with WHO discriminating concentrations of the adulticides for *Ae. aegypti* (Karunaratne et al., 2013; WHO, 2016).

**TABLE 1:** Adulticide susceptibilities of adult female *Aedes aegypti* from laboratory colonies.

Adulticide (concentration*)	Mean per cent mortality ± sd				
	JBW	JFW	NFW	JBWR	JFWR
Deltamethrin (0.03%)	^**c**^88 ± 5	^**a**^95 ± 4	^**a**^97 ± 4	^**a**,**b**^93 ± 3	^**b**,**c**^90 ± 4
Malathion (0.8%)	^**b**^86 ± 4	^**a**^92 ± 4	^**a**^94 ± 4	^**a**^95 ± 3	^**a**^ 91 ± 4
Permethrin, (0.25%)	^**b**^87 ± 3	^**a**^95 ± 2	^**a**^97 ± 4	^**a**^96 ± 4	^**b**^91 ± 4
Propoxur (0.1%)	^**a**^97 ± 4	^**a**^99 ± 2	^**a**^100	^**a**^98 ± 3	^**a**^ 97 ± 4
Etofenprox (0.5%)	^**c**^89 ± 4	^**a**^97 ± 2	^**a**^98 ± 3	^**a**^96 ± 3	^**b**^93 ± 3

*Note*: Number of mosquitoes tested per colony per adulticide was 200. Mosquitoes were exposed to adulticide‐impregnated filter papers for 1 h and mortalities determined after a 24 h recovery period. Values that do not share a letter were significantly different (*p* < 0.05) from others in the same row.

Abbreviation: sd, standard deviation.

* WHO discriminating adulticide concentration (Karunaratne et al., 2013; WHO, 2016).

The bioassays showed that female JBW *Ae. aegypti* were significantly more resistant to the three pyrethroids and malathion, and tended to be more resistant to propoxur than either JFW or NFW females. NFW females with no ancestral exposure to BW in the hill country tended to be more susceptible to all the adulticides than JFW females, but the differences did not achieve statistical significance. JBW females were categorized as resistant to malathion and all three pyrethroids (<90% mortality) and as possibly resistant to propoxur (90%–97% mortality) by WHO criteria for field populations of mosquitoes (Karunaratne et al., 2013; WHO, 2016). In contrast, JFW and NFW females were categorized as susceptible to propoxur (≥98% mortality), NFW as susceptible to etofenprox, JFW as only possibly resistant to etofenprox and both JFW and NFW as only possibly resistant to deltamethrin, malathion and permethrin by the WHO criteria.

The findings from colonies where preimaginal stage salinities were reversed showed that females emerging from reversal colonies tended to alter their adulticide susceptibilities to those corresponding to the new salinities in which the preimaginal stages were reared, that is, the JFWR generally tended to approach that of JBW, and JBWR was not significantly different from JFW *Ae. aegypti*. The reversal of adulticide susceptibility was evident 7–13 generations after changing preimaginal salinity and was more prominent in JBWR than JFWR mosquitoes. Therefore, the salinities to which their preimaginal stages were exposed tended to govern the susceptibilities of adult females to the adulticides.

### Assays for enzymatic insecticide resistance mechanisms in adult female *Aedes aegypti* from JBW and JFW laboratory colonies

Standard WHO protocols (WHO, 1998) were used to investigate whether altered activities of common enzymes known to detoxify adulticides can be responsible for the greater adulticide resistance of JBW females compared to JFW. The results for three major enzyme types are presented in Table 2.

**TABLE 2:** Activities of adulticide detoxifying enzymes in adult female *Aedes aegypti* from JBW and JFW laboratory colonies.

	GST^a^	EST^b^	MO^c^
	Mean activity ± SD (no. of mosquitoes assayed)	Mean activity ± SD (no. of mosquitoes assayed)	Mean activity ± SD (no. of mosquitoes assayed)
JFW (G77)	0.24 ± 0.09 (*n* = 116)	0.10 ± 0.07 (*n* = 100)	0.05 ± 0.03 (*n* = 60)
JBW (G77)	0.24 ± 0.11 (*n* = 116)	0.09 ± 0.05 (*n* = 100)	0.09 ± 0.08 (*n* = 60)
Significance of differences with t‐test value (t) and probability (p)	*t* = −0.44 *p* = 0.66	*t* = −1.10 *p* = 0.27	*t* = 2.85 *p* = 0.005

Abbreviation: *n*, number of mosquitoes assayed.

^a^ Mean glutathione S‐transferase (GST)‐specific activity in micromole per minute per milligram protein with resistance corresponding to >0.40 micromole per minute per milligram protein.

^b^ Mean non‐specific esterase (EST) specific activity in micromole per minute per milligram protein, with resistance corresponding to >0.25 micromole per minute per milligram protein.

^c^ Mean mono‐oxygenase (MO) activity expressed as equivalent units of cytochrome c with resistance corresponding to >0.35 units. Specific activities corresponding to resistance in JBW and JFW females were those previously used to characterize resistance in *Ae. aegypti* field populations in Sri Lanka (Nugapola et al., 2020).

The enzyme assays showed no significant differences in the specific activities of glutathione‐S‐transferases (GSTs) and non‐specific esterases (ESTs) between JBW and JFW females, and a modest increase in the specific activity of monooxygenase (MOs) in JBW females compared with JFW females. The specific activities of GSTs, ESTs and MOs in JBW females were 60%, 36% and 26%, respectively of specific activities ascribed to resistance in field *Ae. aegypti* populations in Sri Lanka (Nugapola et al., 2020).

### Attrition in JBW and JFW *Aedes aegypti* developmental stages on initial reversal of salinity

We estimated the impact of selection caused by reversing salinity during the establishment of the JBWR and JFWR colonies by enumerating the numbers of eggs that hatched into L1 and then developed through L2, L3, L4 and pupa into adults for the G1 to G7 generations (Supplementary Information, Table S5).

Principally in JFWR, and to a lower extent in JBWR, the altered salinity primarily led to attrition in the hatching of eggs into L1 and to a lesser extent in the L1 to L2 transition. In the JBWR colony, the attrition during egg hatching and the L1 to L2 transition observed in G1 tended to stabilize in G2 and successive generations. Attrition during the hatching of eggs and the L1 to L2 transition in the JFWR colony was more marked than in the JBWR colony in G1, progressively diminished in subsequent generations, and tended to stabilize only in G7.

### Morphological features of adult female *Aedes aegypti* from the JBW and JFW laboratory colonies

Visual inspection did not reveal detectable differences between adult females from the JBW and JFW colonies. The body wet weights of 20 sugar‐fed, 3‐day‐old females from the G84 JBW and G84 JFW colonies (JBW = 1.32 ± 0.20 mg; JFW = 1.35 ± 0.19 mg; mean ± standard deviation) were not significantly different (t = 0.47, *p* = 0.64). The wing lengths of the same mosquitoes (JBW = 2.40 ± 0.09 mm; JFW = 2.38 ± 0.10 mm; mean ± standard deviation), which are surrogates for mosquito sizes (Nasci, 1986), were also not significantly different (t = 0.43, *p* = 0.35).

## DISCUSSION

Adulticide resistance in *Aedes* field populations selected by the widespread use of adulticides for mosquito vector control is an increasing worldwide problem for controlling arboviral diseases (Karunaratne et al., 2013; Marcombe et al., 2009; Sumitha et al., 2023; Vontas et al., 2020; WHO, 2023). Well‐established resistance mechanisms include increased activities of the adulticide‐detoxifying enzyme families ESTs, GSTs and cytochrome P450‐containing MOs (CYPs), as well as mutations in protein targets of adulticides such as acetylcholinesterase and the voltage‐gated sodium channel protein (VGSC) (Hemingway et al., 2004; Karunaratne et al., 2013; Liu, 2015; Marcombe et al., 2009; Sumitha et al., 2023; Vontas et al., 2020; WHO, 2023). The *Ae. aegypti* genome contains multiple genes for ESTs, GSTs and MOs (Strode et al., 2008). The findings that EST and GST activities are not different between JBW and JFW females show that increased activities of the two enzyme families are not responsible for the greater adulticide resistance in JBW females. CYPs play an important role in detoxifying type 1 and 2 pyrethroids and are also able to detoxify carbamate and organophosphate adulticides (Vontas et al., 2020). The modestly greater MO activity in JBW adult females was only 26% of the value ascribed to resistance in field populations of *Ae. aegypti* (Nugapola et al., 2020) and might be solely due to the induction of CYPs needed for the increased synthesis of long‐chain hydrocarbons in the epicuticle, as discussed below. Reversal of the greater resistance to the pyrethroids and malathion in JBWR females within 7–13 generations in fresh water is inconsistent with mutations in the coding sequences for VGSC and acetylcholinesterase being respectively responsible for the greater resistance in JBW females. DNA sequence analysis for relevant mutations to confirm this could not be undertaken during the present study.

Cuticle changes are another cause of the adulticide resistance selected through exposure to adulticides in several mosquito species (Balabanidou et al., 2016 & 2019; Bass & Jones, 2016; Hemingway et al., 2004; Huang et al., 2018; Jacobs et al., 2023; Liu, 2015; Wood et al., 2010; Xu et al., 2020; Xu et al., 2022), the oriental fruit fly *Bactrocera dorsalia* (Lin et al., 2012), the bed bug *Cimex lectularius* (Lily et al., Lilly et al., 2016) and the cockroach *Blattella germanica* (Cai et al., 2024). A comparison of pyrethroid‐resistant and ‐susceptible FW laboratory strains of *Ae. aegypti* reported that resistant‐strain adults possessed thicker procuticles in femurs and higher cuticle polysaccharide content (Jacobs et al., 2023). Thicker cuticles in T1 correlated with permethrin resistance in FW laboratory strains of the malaria vector *Anopheles funestus* (Wood et al., 2010). Cuticle thickening in femurs (Balabanidou et al., 2016) and tarsi (Balabanidou et al., 2019) of FW laboratory strains of *Anopheles gambiae*, a major African malaria vector, also correlated with adulticide resistance. The tendency for chitinaceous lamellae to be more prominent within the thicker abdomen exocuticles of BW‐developing females that we observed here is consistent with the higher polysaccharide content reported in the thicker cuticles of adulticide‐resistant *Ae. aegypti* (Jacobs et al., 2023) and the higher chitin and chitin‐binding protein content in the thicker cuticles of adulticide‐resistant *An. gambiae* (Balabanidou et al., 2016 & 2019).

We showed here for the first time that *Ae. aegypti* preimaginal stages developing in BW of 10 g/L salt in the JBW colony and 6–8 g/L salt BW in field habitats produce females with thicker procuticles, as well as the component exocuticles and endocuticles, in T1 tarsomeres and abdomens than females from the JFW colony that underwent preimaginal development in FW. Female *Ae. aegypti* derived from BW‐developing preimaginal stages also tended to have more prominent lamellae in the exocuticles of abdomens than females from the JFW colony. The 26% greater thickness of T1 tarsomere procuticles in JBW and BW field compared with JFW *Ae. aegypti* that we observed lies within the range of the procuticle thickening reported for pyrethroid‐resistant *An. funestus* T1 of 9% (Wood et al., 2010) and pyrethroid‐resistant *An. gambiae* tarsi of 52% (Balabanidou et al., 2019) compared to the corresponding susceptible strains. T1 and abdomen procuticle structures of JBW and JFW female *Ae. aegypti* either reversed or tended to reverse when the preimaginal salinities were respectively reversed in the JBWR and JFWR laboratory colonies, suggesting that it is the salinity to which the preimaginal stages are exposed that influences procuticle structure in the ensuing adults. The marked reversal of procuticle thicknesses within 10 generations to that characteristic of JFW females, when JBW are transferred to FW in the JBWR colony, also shows that irreversible genetic differences are not responsible for the thicker procuticles in JBW *Ae. aegypti*. TEM previously showed that procuticles were thicker and chitinaceous lamellae tended to be more prominent in JBW L4 compared with JFW L4 (Ramasamy et al., 2021). Furthermore, atomic force microscopy showed that JBW L3 and L4 had rougher cuticle surfaces than the corresponding JFW larvae (Sivabalakrishnan et al., 2023). Taken together, these findings imply that procuticle changes in BW‐developing larvae may be carried through to the adult stages of *Ae. aegypti*. Differences in procuticle ultrastructure of T1 tarsomeres and abdomens of adult females that we observed here are likely to be caused by the different ontogeny of the two mosquito structures.

Importantly, cuticle changes in T1 tarsi and abdomens of JBW females were accompanied by increased resistance, when compared with females from two different FW colonies, to three common classes of adulticides in bioassays that depend on tarsal contact with the adulticides (Karunaratne et al., 2013; WHO, 2016). That the greater adulticide resistance in JBW *Ae. aegypti* is due to preimaginal salinity‐induced changes in T1 procuticles is supported by the tendency for both adulticide resistance and T1 procuticle structure to change to those of JFW *Ae. aegypti* in the JBWR colony where *Ae. aegypti* from the JBW colony were reared in FW. The structural changes in the abdominal procuticles of female *Ae. aegypti* undergoing preimaginal development in BW are also relevant because, although not tested here, they can potentially confer greater resistance to adulticides used as space sprays to control epidemics of arboviral diseases (WHO, 2009a). The reversal of procuticle structures in both T1 and abdomen as well as adulticide resistance was more obvious in the JBWR colony than the JFWR colony. This is consistent with the attrition data showing that BW‐developing *Ae. aegypti* more readily adapt to FW than vice versa, that is, adaptation of FW‐developing *Ae. aegypti* to BW involves greater selection pressure than vice versa. The present findings with adult *Ae. aegypti* are supported by observations that JBW L4 with thicker and remodelled procuticles (Ramasamy et al., 2021) were more resistant than JFW L4 to the larvicide temephos (Sivabalakrishnan et al., 2023). Reversal of salinity in JBWR and JFWR colonies showed that preimaginal rearing salinity was the determining factor for the differential temephos resistance in L4 (Sivabalakrishnan et al., 2023). A causal relationship between procuticle thickening and the greater resistance to adulticides in JBW females may also be inferred by analogy with findings from other insects where resistance selected by exposure to adulticides has been associated with cuticle thickening, for example, in *Ae. aegypti* (Jacobs et al., 2023), *An. funestus* (Wood et al., 2010), *An. gambiae* (Balabanidou et al., 2016 & 2019), *Culex pipiens* (Huang et al., 2018; Xu et al., 2020; Xu et al., 2022), *B. dorsalis* (Lin et al., 2012), *C. lectularius* (Lilly et al., 2016) and *B. germanica* (Cai et al., 2024), with a role also for epicuticle changes as discussed below. The mechanism underlying adulticide‐induced resistance in *An. gambiae* (Balabanidou et al., 2016) and *B. dorsalis* (Lin et al., 2012) was shown to be the decreased penetration of adulticides through the thicker cuticles.

Molecular mechanisms underlying the procuticle changes in adulticide‐resistant mosquitoes are beginning to emerge. RNA interference (RNAi) experiments identified CPR63 (Xu et al., 2022), a member of the RR‐2 family of cuticle proteins (Willis, 2010) and CPLCG5 (Huang et al., 2018), a member of the CPLC cuticle protein family (Willis, 2010), as essential for forming the thicker procuticles associated with pyrethroid‐selected resistance in adult *Cx. pipiens*. It is pertinent that many RR‐2 and CPLCG proteins detected in the thicker cuticles of JBW L4 were absent in JFW L4 cuticles, and that transcripts for many RR‐2 cuticle proteins were highly upregulated in JBW L4 compared with JFW L4 (Ramasamy et al., 2021). Expression of the *cplcg5* gene is controlled by the transcription factor Fushi tarazu‐Factor 1 (FTZ‐F1), which was upregulated in pyrethroid‐resistant adult *Cx. pipiens* (Xu et al., 2020). Transcripts for FTZ‐F1 were also markedly upregulated in JBW L4 compared with JFW L4 (Ramasamy et al., 2021). Continuing greater expression of these genes in pupae and adults might contribute to the procuticle structural changes associated with greater adulticide resistance in JBW females.

The insect epicuticle is an important permeability barrier that is rich in very long‐chain hydrocarbons and waxy esters (Richards, 1978). There is evidence to suggest that epicuticle modifications, in addition to the procuticle changes, may contribute to the greater adulticide resistance in JBW females. Biosynthesis of the very long‐chain hydrocarbons requires considerable elongation of fatty acid acyl CoA, subsequent reduction by a NADH/NADPH‐dependent fatty acid acyl CoA reductase to an aldehyde, followed by decarbonylation catalysed by an insect‐specific NADPH‐dependent CYP enzyme of the 4G1family that was first characterized in *Drosophila* (Qiu et al., 2012). *Drosophila* null mutants of *cyp4g1* died rapidly after emerging from pupae, suggesting that very long‐chain hydrocarbons were essential for adults to withstand desiccation (Qiu et al., 2012). Two homologues of *Drosophila cyp4g1* present in *An. gambiae*, termed *cyp4g16* and *cyp4g17*, encode decarbonylases producing very long‐chain hydrocarbons (Balabanidou et al., 2016 & 2019; Kefi et al., 2019). *Cyp4g16* and *cyp4g17* were overexpressed in pyrethroid‐resistant adult *An. gambiae* that were also characterized by (i) thicker epicuticles and procuticles, (ii) elevated cuticular hydrocarbons, chitin and chitin‐binding cuticular protein content in legs of adult females and (iii) reduced internalization of topically applied deltamethrin (Balabanidou et al., 2016 & 2019). *Aedes aegypti* genes with closest homologies to *An. gambiae cyp4g16* and *cyp4g17* are AAEL004054 (88% coding protein identity to *An. gambiae* CYP4G16) and AAEL006804 (81% protein identity to the available partial sequence of *An. gambiae* CYP4G17) respectively. Their transcripts were more abundant in JBW L4 than JFW L4 (Ramasamy et al., 2021), and if carried through to adults can cause the higher MO activity observed in JBW females. Compared with JFW L4, transcripts for fatty acid synthase, very long‐chain fatty acid elongase and fatty acyl CoA reductase also showed large increases in JBW L4 (Ramasamy et al., 2021). Increased amounts of very long‐chain cuticular hydrocarbons in the epicuticle, as well as the differential synthesis of chitin and cuticle proteins in the procuticle, were proposed to minimize the osmotic efflux of water and influx of ions (Ramasamy et al., 2021), and lead to greater resistance to the larvicide temephos through reduced uptake in JBW L4 (Sivabalakrishnan et al., 2023). The same epicuticle and procuticle changes expressed in JBW females may be responsible for their greater adulticide resistance in an analogous manner to adulticide‐resistant *An. gambiae* (Balabanidou et al., 2016 & 2019). Investigations on the rates of penetration of adulticides through the cuticle, which were not possible in the present study, can help to clarify mechanisms underlying the differential adulticide resistance in JBW and JFW *Ae. aegypti*.

Our findings show that attrition in JFW *Ae. aegypti* transferred to 10 g/L salt BW occurs principally during egg hatching and early larval development and requires several generations to stabilize. Furthermore, salinity‐adaptive ultrastructural changes in the procuticle and greater adulticide resistance in JBW females are reversed on transfer to FW in the JBWR colony, which is also consistent with the reversal of the greater temephos resistance in JBW L3 and L4 transferred to FW (Sivabalakrishnan et al., 2023; Supplementary Information, Table S6). Additionally, genetic crosses between JBW and JFW males and females showed that greater hatchability of F1 eggs in BW was a maternally inherited property (Sivabalakrishnan et al., 2023). Environmental stressors other than salinity that cause delayed hatching of eggs are known to produce phenotypic changes in adult *Ae. aegypti* (Perez & Noriega, 2013). The different findings discussed here may be brought together in an experimentally testable hypothesis that salinity‐induced selection in FW *Ae. aegypti* during egg hatching and early larval development in BW leads to inheritable epigenetic changes expressed in larvae and adults of BW *Ae. aegypti* that manifest as (i) greater hatchability of eggs in BW, (ii) greater tolerance of preimaginal stages to salt influx and water efflux in BW due to changes in the epicuticle and procuticle, (iii) reduced susceptibility to larvicides and adulticides in BW *Ae. aegypti* due to the same epicuticle and procuticle changes and (iv) reversibility of the epigenetic and phenotypic changes over several generations when the salinity‐selection pressure is removed by transferring BW *Ae. aegypti* to FW.

Similar BW‐adaptive changes, including diminished larvicide and adulticide susceptibility, can potentially occur in other FW mosquito vectors that have been recently shown to be capable of developing in coastal BW collections, such as *Ae. albopictus*, *Anopheles culicifacies* and *Anopheles stephensi* (Jude et al., 2010 & 2012; Ramasamy et al., 2011; Surendran, Sivabalakrishnan, et al., 2019). BW‐developing *Ae. aegypti* and *Ae. albopictus* can be infected with the dengue virus and transovarially transmit the virus (Surendran et al., 2018), and hence are able to function as virus reservoirs in the absence of vector control measures targeting BW habitats in coastal areas (Ramasamy, 2024; Surendran et al., 2018). There is also a potential for FW *Ae. aegypti* adapting to BW to evolve into a new species in geographically isolated coastal locations mirroring, for example, the evolution of the BW‐tolerant malaria vector *Anopheles merus* from FW *An. gambiae* in Eastern Africa (O'Loughlin et al., 2014).

Our findings highlight the importance of (i) extending mosquito vector control measures that presently focus on FW preimaginal habitats (US CDC, 2023; WHO, 2009a, 2009b) to coastal BW habitats (Ramasamy, 2024), and (ii) monitoring larvicide and adulticide resistance in BW‐developing mosquito vectors and optimizing concentrations of insecticides for use against BW vectors. This may be particularly important in the context of the BW habitats of salinity‐tolerant mosquito vectors increasing as a result of rising sea levels caused by global warming and the accumulation of littoral container debris able to hold BW (Ramasamy, 2024; Ramasamy & Surendran, 2011, 2012, 2016; Surendran et al., 2020).

## AUTHOR CONTRIBUTIONS

**Kokila Sivabalakrishnan:** Investigation; writing – review and editing. **Andrew Hemphill:** Investigation; writing – review and editing; project administration; supervision; funding acquisition. **S. H. P. Parakrama Karunaratne:** Supervision; writing – review and editing. **Arunasalam Naguleswaran:** Supervision; writing – review and editing; funding acquisition. **Isabel Roditi:** Funding acquisition; writing – review and editing. **Sinnathamby N. Surendran:** Conceptualization; project administration; supervision; funding acquisition; formal analysis; investigation. **Ranjan Ramasamy:** Conceptualization; investigation; writing – original draft; formal analysis; supervision; writing – review and editing.

## CONFLICT OF INTEREST STATEMENT

The authors declare no conflicts of interest.

## ETHICS STATEMENT

Mosquitoes were collected and reared with approval from the Institutional Animal Ethics Committee of the University of Jaffna AERC/2023/03v3.

## Supporting information


**Data S1.** Supporting Information.

## Data Availability

All data necessary for the conclusions are included in the manuscript and its Supporting Information. The Supporting Information is publicly accessible and deposited in the Data Repository of the Department of Zoology, University of Jaffna at https://www.zoo.jfn.ac.lk/data-repository and the file is labelled MVE 2025. [Correction added on 26 September 2025 after first online publication: The Data Availability Statement has been updated.]

## References

[mve12799-bib-0001] Balabanidou, V. , Kampouraki, A. , MacLean, M. , Blomquist, G.J. , Tittiger, C. , Juárez, M.P. et al. (2016) Cytochrome P450 associated with insecticide resistance catalyzes cuticular hydrocarbon production in *Anopheles gambiae* . Proceedings of the National Academy of Sciences of the United States of America, 113(33), 9268–9273. Available from: 10.1073/pnas.1608295113 PMC4995928 27439866

[mve12799-bib-0002] Balabanidou, V. , Kefi, M. , Aivaliotis, M. , Koidou, V. , Girotti, J.R. , Mijailovsky, S.J. et al. (2019) Mosquitoes cloak their legs to resist insecticides. Proceedings of the Royal Society B: Biological Sciences, 286(1907), 20191091. Available from: 10.1098/rspb.2019.1091 PMC6661348 31311476

[mve12799-bib-0003] Bass, C. & Jones, C.M. (2016) Mosquitoes boost body armor to resist insecticide attack. Proceedings of the National Academy of Sciences of the United States of America, 113(33), 9145–9147. Available from: 10.1073/pnas.1610992113 PMC4995925 27496323

[mve12799-bib-0004] Bradley, T.J. (1987) Physiology of osmoregulation in mosquitoes. Annual Review of Entomology, 32(1), 439–462. Available from: 10.1146/annurev.en.32.010187.002255 2880552

[mve12799-bib-0005] Cai, T. , Wang, X. , Liu, B. , Zhao, H. , Liu, C. , Zhang, X. et al. (2024) A cuticular protein, BgCPLCP1, contributes to insecticide resistance by thickening the cockroach endocuticle. International Journal of Biological Macromolecules, 254(Pt 1), 127642. Available from: 10.1016/j.ijbiomac.2023.127642 37898258

[mve12799-bib-0006] Hemingway, J. , Hawkes, N.J. , McCarroll, L. & Ranson, H. (2004) The molecular basis of insecticide resistance in mosquitoes. Insect Biochemistry and Molecular Biology, 34(7), 653–665. Available from: 10.1016/j.ibmb.2004.03.018 15242706

[mve12799-bib-0007] Huang, Y. , Guo, Q. , Sun, X. , Zhang, C. , Xu, N. , Xu, Y. et al. (2018) *Culex pipiens pallens* cuticular protein CPLCG5 participates in pyrethroid resistance by forming a rigid matrix. Parasites & Vectors, 11(1), 6. Available from: 10.1186/s13071-017-2567-9 PMC5753453 29301564

[mve12799-bib-0008] Jacobs, E. , Chrissian, C. , Rankin‐Turner, S. , Wear, M. , Camacho, E. , Broderick, N.A. et al. (2023) Cuticular profiling of insecticide resistant *Aedes aegypti* . Scientific Reports, 13(1), 10154. Available from: 10.1038/s41598-023-36926-3 PMC10287657 37349387

[mve12799-bib-0009] Jude, P.J. , Dharshini, S. , Vinobaba, M. , Surendran, S.N. & Ramasamy, R. (2010) *Anopheles culicifacies* breeding in brackish waters in Sri Lanka and implications for malaria control. Malaria Journal, 9, 106. Available from: 10.1186/1475-2875-9-106 PMC2864285 20409313

[mve12799-bib-0010] Jude, P.J. , Tharmasegaram, T. , Sivasubramaniyam, G. , Senthilnanthanan, M. , Kannathasan, S. , Raveendran, R. et al. (2012) Salinity‐tolerant larvae of mosquito vectors in the tropical coast of Jaffna, Sri Lanka and the effect of salinity on the toxicity of *Bacillus thuringiensis* to *Aedes aegypti* larvae. Parasites & Vectors, 5, 269. Available from: 10.1186/1756-3305-5-269 PMC3533938 23174003

[mve12799-bib-0011] Karunaratne, S.H.P.P. , Weeraratne, T.C. , Perera, M.D.B. & Surendran, S.N. (2013) Insecticide resistance and efficacy of space spraying and larviciding in the control of dengue vectors *Aedes aegypti* and *Aedes albopictus* in Sri Lanka. Pesticide Biochemistry and Physiology, 107, 98–105. Available from: 10.1016/j.pestbp.2013.05.011 25149242

[mve12799-bib-0012] Kefi, M. , Balabanidou, V. , Douris, V. , Lycett, G. , Feyereisen, R. & Vontas, J. (2019) Two functionally distinct CYP4G genes of *Anopheles gambiae* contribute to cuticular hydrocarbon biosynthesis. Insect Biochemistry and Molecular Biology, 110, 52–59. Available from: 10.1016/j.ibmb.2019.04.018 31051237

[mve12799-bib-0013] Lilly, D.G. , Latham, S.L. , Webb, C.E. & Doggett, S.L. (2016) Cuticle thickening in a pyrethroid‐resistant strain of the common bed bug, *Cimex lectularius* L. (Hemiptera: Cimicidae). PLoS One, 11(4), e0153302. Available from: 10.1371/journal.pone.0153302 PMC4830598 27073871

[mve12799-bib-0014] Lin, Y. , Jin, T. , Zeng, L. & Lu, Y. (2012) Cuticular penetration of β‐cypermethrin in insecticide‐susceptible and resistant strains of *Bactrocera dorsalis* . Pesticide Biochemistry and Physiology, 103, 189–193. Available from: 10.1016/j.pestbp.2012.05.002

[mve12799-bib-0015] Liu, N. (2015) Insecticide resistance in mosquitoes: impact, mechanisms, and research directions. Annual Review of Entomology, 7, 537–559. Available from: 10.1146/annurev-ento-010814-020828 25564745

[mve12799-bib-0016] Marcombe, S. , Poupardin, R. , Darriet, F. , Reynaud, S. , Bonnet, J. , Strode, C. et al. (2009) Exploring the molecular basis of insecticide resistance in the dengue vector *Aedes aegypti*: a case study in Martinique Island (French West Indies). BMC Genomics, 10, 494. Available from: 10.1186/1471-2164-10-494 PMC2770535 19857255

[mve12799-bib-0017] Moussian, B. (2010) Recent advances in understanding mechanisms of insect cuticle differentiation. Insect Biochemistry and Molecular Biology, 40, 363–375. Available from: 10.1016/j.ibmb.2010.03.003 20347980

[mve12799-bib-0018] Nasci, R.S. (1986) The size of emerging and host‐seeking *Aedes aegypti* and the relation of size to blood‐feeding success in the field. Journal of the American Mosquito Control Association, 2(1), 61–62. 3507471

[mve12799-bib-0019] Nugapola, N.W.N.P. , de Silva, W.A.P.P. , Weeraratne, T.C. & Karunaratne, S.H.P.P. (2020) *Kdr* type mutations and enhanced GST based insecticide resistance in dengue vector mosquitoes *Aedes aegypti* and *Aedes albopictus* . International Journal of Tropical Insect Science, 41(2), 409–417. Available from: 10.1007/s42690-020-00219-3

[mve12799-bib-0020] O'Loughlin, S.M. , Magesa, S. , Mbogo, C. , Mosha, F. , Midega, J. , Lomas, S. et al. (2014) Genomic analyses of three malaria vectors reveals extensive shared polymorphism but contrasting population histories. Molecular Biology and Evolution, 31(4), 889–902. Available from: 10.1093/molbev/msu040 PMC3969563 24408911

[mve12799-bib-0021] Perez, M.H. & Noriega, F.G. (2013) *Aedes aegypti* pharate 1st instar quiescence: a case for anticipatory reproductive plasticity. Journal of Insect Physiology, 59(3), 318–324. Available from: 10.1016/j.jinsphys.2012.12.007 PMC3596445 23298690

[mve12799-bib-0022] Qiu, Y. , Tittiger, C. , Wicker‐Thomas, C. , Le Goff, G. , Young, S. , Wajnberg, E. et al. (2012) An insect‐specific P450 oxidative decarbonylase for cuticular hydrocarbon biosynthesis. Proceedings of the National Academy of Sciences USA, 109(37), 14858–14863. Available from: 10.1073/pnas.1208650109 PMC3443174 22927409

[mve12799-bib-0023] Ramasamy, R. (2024) Analysis of some societal factors constraining COVID‐19, dengue, malaria and tick‐borne borreliosis control. Archives of Microbiology and Immunology, 8, 365–375. Available from: 10.26502/ami.936500183

[mve12799-bib-0024] Ramasamy, R. , Jude, P.J. , Veluppillai, T. , Eswaramohan, T. & Surendran, S.N. (2014) Biological differences between brackish and fresh water‐derived *Aedes aegypti* from two locations in the Jaffna peninsula of Sri Lanka and the implications for arboviral disease transmission. PLoS One, 9, e104977. Available from: 10.1371/journal.pone.0104977 PMC4149417 25170879

[mve12799-bib-0025] Ramasamy, R. & Surendran, S.N. (2011) Possible impact of rising sea levels on vector‐borne infectious diseases. BMC Infectious Diseases, 11, 18. Available from: 10.1186/1471-2334-11-18 PMC3035583 21241521

[mve12799-bib-0026] Ramasamy, R. & Surendran, S.N. (2012) Global climate change and its potential impact on disease transmission by salinity‐tolerant mosquito vectors in coastal zones. Frontiers in Physiology, 3, 198. Available from: 10.3389/fphys.2012.00198 PMC3377959 22723781

[mve12799-bib-0027] Ramasamy, R. & Surendran, S.N. (2016) Mosquito vectors developing in atypical anthropogenic habitats: global overview of recent observations, mechanisms and impact on disease transmission. Journal of Vector Borne Diseases, 53(2), 91–98. 27353577

[mve12799-bib-0028] Ramasamy, R. , Surendran, S.N. , Jude, P.J. , Dharshini, S. & Vinobaba, M. (2011) Larval development of *Aedes aegypti* and *Aedes albopictus* in peri‐urban brackish water and its implications for transmission of arboviral diseases. PLoS Neglected Tropical Diseases, 5, e1369. Available from: 10.1371/journal.pntd.0001369 PMC3222631 22132243

[mve12799-bib-0029] Ramasamy, R. , Thiruchenthooran, V. , Jayadas, T.T.P. , Eswaramohan, T. , Santhirasegaram, S. , Sivabalakrishnan, K. et al. (2021) Transcriptomic, proteomic and ultrastructural studies on salinity‐tolerant *Aedes aegypti* in the context of rising sea levels and arboviral disease epidemiology. BMC Genomics, 22, 253. Available from: 10.1186/s12864-021-07564-8 PMC8034070 33836668

[mve12799-bib-0030] Richards, G. (1978) In: Rockstein, M. (Ed.) The chemistry of insect cuticle in biochemistry of insects. New York: Academic, pp. 205–232.

[mve12799-bib-0031] Rueda, L.M. (2004) Pictorial keys for the identification of mosquitoes (Diptera: Culicidae) associated with dengue virus transmission. Zootaxa, 589(1), 1. Available from: 10.11646/ZOOTAXA.589.1.1

[mve12799-bib-0032] Schindelin, J. , Arganda‐Carreras, I. , Frise, E. , Kaynig, V. , Longair, M. , Pietzsch, T. et al. (2012) Fiji: an open‐source platform for biological‐image analysis. Nature Methods, 9(7), 676–682. Available from: 10.1038/nmeth.2019 PMC3855844 22743772

[mve12799-bib-0033] Sivabalakrishnan, K. , Thanihaichelvan, M. , Tharsan, A. , Eswaramohan, T. , Ravirajan, P. , Hemphill, A. et al. (2023) Resistance to the larvicide temephos and altered egg and larval surfaces characterize salinity‐tolerant *Aedes aegypti* . Scientific Reports, 13(1), 8160. Available from: 10.1038/s41598-023-35128-1 PMC10198600 37208485

[mve12799-bib-0034] Strode, C. , Wondji, C.S. , David, J.P. , Hawkes, N.J. , Lumjuan, N. , Nelson, D.R. et al. (2008) Genomic analysis of detoxification genes in the mosquito *Aedes aegypti* . Insect Biochemistry and Molecular Biology, 38(1), 113–123. Available from: 10.1016/j.ibmb.2007.09.007 18070670

[mve12799-bib-0035] Sumitha, M.K. , Kalimuthu, M. , Senthil, M.K. , Paramasivan, R. , Kumar, A. & Gupta, B. (2023) Status of insecticide resistance in the dengue vector *Aedes aegypti* in India: a review. Journal of Vector Borne Diseases, 60(2), 116–124. Available from: 10.4103/0972-9062.361174 37417161

[mve12799-bib-0036] Surendran, S.N. , Jayadas, T.T.P. , Sivabalakrishnan, K. , Santhirasegaram, S. , Karvannan, K. , Weeraratne, T.C. et al. (2019) Development of the major arboviral vector *Aedes aegypti* in urban drain‐water and associated pyrethroid insecticide resistance is a potential global health challenge. Parasites & Vectors, 12(1), 337. Available from: 10.1186/s13071-019-3590-9 PMC6615112 31287014

[mve12799-bib-0037] Surendran, S.N. , Jayadas, T.T.P. , Thiruchenthooran, V. , Raveendran, S. , Tharsan, A. , Santhirasegaram, S. et al. (2021) *Aedes* larval bionomics and implications for dengue control in the paradigmatic Jaffna peninsula, northern Sri Lanka. Parasites & Vectors, 14, 162. Available from: 10.1186/s13071-021-04640-6 PMC7977581 33736702

[mve12799-bib-0038] Surendran, S.N. , Jude, P.J. , Thabothiny, V. , Raveendran, S. & Ramasamy, R. (2012) Preimaginal development of *Aedes aegypti* in brackish and fresh water urban domestic wells in Sri Lanka. Journal of Vector Ecology, 37(2), 471–473. Available from: 10.1111/j.1948-7134.2012.00254.x 23181875

[mve12799-bib-0039] Surendran, S.N. , Nagulan, R. , Sivabalakrishnan, K. , Arthiyan, S. , Tharsan, A. , Jayadas, T.T.P. et al. (2022) Reduced dengue incidence during the COVID‐19 movement restrictions in Sri Lanka from march 2020 to April 2021. BMC Public Health, 22(1), 388. Available from: 10.1186/s12889-022-12726-8 PMC8866919 35209890

[mve12799-bib-0040] Surendran, S.N. , Nagulan, R. , Tharsan, A. , Sivabalakrishnan, K. & Ramasamy, R. (2022) Dengue incidence and *Aedes* vector collections in relation to COVID‐19 population mobility restrictions. Tropical Medicine and Infectious Diseases, 7(10), 287. Available from: 10.3390/tropicalmed7100287 PMC9612376 36288027

[mve12799-bib-0041] Surendran, S.N. , Senthilnanthanan, M. , Jayadas, T.T.P. , Karunaratne, S.H.P.P. & Ramasamy, R. (2020) Impact of salinization and pollution of groundwater on the adaptation of mosquito vectors in the Jaffna peninsula, Sri Lanka. Ceylon Journal of Science, 49(2), 135–150. Available from: 10.4038/cjs.v49i2.7734

[mve12799-bib-0042] Surendran, S.N. , Sivabalakrishnan, K. , Sivasingham, A. , Jayadas, T.T.P. , Karvannan, K. , Santhirasegaram, S. et al. (2019) Anthropogenic factors driving recent range expansion of the malaria vector *Anopheles stephensi* . Frontiers in Public Health, 7, 53. Available from: 10.3389/fpubh.2019.00053 PMC6426791 30923705

[mve12799-bib-0043] Surendran, S.N. , Veluppillai, T. , Eswaramohan, T. , Sivabalakrishnan, K. , Noordeen, F. & Ramasamy, R. (2018) Salinity tolerant *Aedes aegypti* and *Ae. Albopictu*s‐infection with dengue virus and contribution to dengue transmission in a coastal peninsula. Journal of Vector Borne Diseases, 55(1), 26–33. Available from: 10.4103/0972-9062.234623 29916445

[mve12799-bib-0044] US Centers for Disease Control and Prevention . (2023) Dengue. Available from: https://www.cdc.gov/dengue/index

[mve12799-bib-0045] Vontas, J. , Katsavou, E. & Mavridis, K. (2020) Cytochrome P450‐based metabolic insecticide resistance in *Anopheles* and *Aedes* mosquito vectors: muddying the waters. Pesticide Biochemistry and Physiology, 170, 104666. Available from: 10.1016/j.pestbp.2020.104666 32980073

[mve12799-bib-0046] Willis, J.H. (2010) Structural cuticular proteins from arthropods: annotation, nomenclature, and sequence characteristics in the genomics era. Insect Biochemistry and Molecular Biology, 40(3), 189–204. Available from: 10.1016/j.ibmb.2010.02.001 PMC2872936 20171281

[mve12799-bib-0047] Wood, O. , Hanrahan, S. , Coetzee, M. , Koekemoer, L. & Brooke, B. (2010) Cuticle thickening associated with pyrethroid resistance in the major malaria vector *Anopheles funestus* . Parasites & Vectors, 3, 67. Available from: 10.1186/1756-3305-3-67 PMC2924294 20684757

[mve12799-bib-0048] World Health Organization . (1998) Techniques to detect insecticide resistance mechanisms (field and laboratory manual). Available from: https://apps.who.int/iris/handle/10665/83780

[mve12799-bib-0049] World Health Organization . (2009a) Dengue guidelines for diagnosis, treatment, prevention and control. Available from: https://whqlibdoc.who.int/publications/2009/9789241547871_eng.pdf 23762963

[mve12799-bib-0050] World Health Organization . (2009b) Temephos in drinking‐water: use for vector control in drinking‐water sources and containers (Tech. Rep. WHO/HSE/WSH/09.01/1). Available from: https://www.who.int/docs/default‐source/wash‐documents/wash‐chemicals/temephos‐background‐document.pdf?sfvrsn=c34fda71_4

[mve12799-bib-0051] World Health Organization . (2016) Monitoring and managing insecticide resistance in *Aedes* mosquito populations. “Interim guidance for entomologists” (Tech. Rep. WHO/ZIKV/VC/16.1). Available from: https://apps.who.int/iris/handle/10665/204588

[mve12799-bib-0052] World Health Organization . (2023) Report on insecticide resistance in *Aedes* mosquitoes (*Aedes aegypti*, *Ae. albopictus*, *Ae. vittatus*) in WHO South‐East Asia Region countries. Available from: https://www.who.int/publications/i/item/sea-cd-334

[mve12799-bib-0053] World Health Organization . (2024) Dengue and severe dengue. Available from: https://www.who.int/news‐room/fact‐sheets/detail/dengue‐and‐severe‐dengue

[mve12799-bib-0054] Xu, Y. , Xu, J. , Zhou, Y. , Li, X. , Meng, Y. , Ma, L. et al. (2022) CPR63 promotes pyrethroid resistance by increasing cuticle thickness in *Culex pipiens pallens* . Parasites & Vectors, 15(1), 54. Available from: 10.1186/s13071-022-05175-0 PMC8842966 35164827

[mve12799-bib-0055] Xu, Y. , Yang, X. , Sun, X. , Li, X. , Liu, Z. , Yin, Q. et al. (2020) Transcription factor FTZ‐F1 regulates mosquito cuticular protein CPLCG5 conferring resistance to pyrethroids in *Culex pipiens pallens* . Parasites & Vectors, 13(1), 514. Available from: 10.1186/s13071-020-04383-w PMC7559895 33054862

