# Supplementary material for: Preimaginal development of Aedes aegypti L. (Diptera: Culicidae) in brackish water gives rise to adult mosquitoes with thicker cuticles and greater insecticide resistance
Source: Med Vet Entomol. 2025 Mar 7;40(4):759–73. doi: 10.1111/mve.12799 (PMC13749582; doi:10.1111/mve.12799)
Supplement: Supplementary file 1 [file MVE-40-759-s001.docx]

Supporting Information

**Preimaginal development of *Aedes aegypti* L. (Diptera: Culicidae) in brackish water gives rise to adult mosquitoes with thicker cuticles and greater insecticide resistance**

Kokila Sivabalakrishnan, Andrew Hemphill, S.H.P. Parakrama Karunaratne, Arunasalam Naguleswaran, Isabel Roditi, Sinnathamby N. Surendran^*^, Ranjan Ramasamy^*^

Corresponding authors: rjr200911@yahoo.com, noble@univ.jfn.ac.lk

**Table S1. Other countries where *Aedes aegypti* and *Aedes albopictus* have been shown to develop in brackish water habitats in the field**

| **Country** | **Species** | **Habitats** | **Reference** |
| --- | --- | --- | --- |
| Brazil | *Ae. aegypti* and *Ae. albopictus* | Discarded household containers, used tyres, water tanks, drains and phytotelmata with BW up to 13.5 g/L salt for *Ae. aegypti* and BW up to 9.2 g/L salt for *Ae. albopictus* in coastal areas of Sao Paulo state. | de Brito Arduino et al., 2010 |
| Brunei Darussalam | *Ae. albopictus* | Discarded plastic containers and empty coconut shells with up to 8 g/L salt BW in beaches and estuarine villages | Idris et al., 2013 |
| India | *Ae. albopictus* | Shallow ponds, plastic containers and discarded bottles with up to 4.5 g/L salt BW in coastal areas of Kerala state | Shamna et al., 2022 |
| Indonesia | *Ae. aegypti* and *Ae. albopictus* | Discarded household containers, used tyres, abandoned boats and wells with up to 5 g/L salt BW in coastal beach areas of Sulawesi | Ratnasari et al., 2020 |

**Legend to Table S1.** BW: brackish water.

| **Table S2. Differences between brackish and fresh water developing *Ae. aegypti* larvae** | | |
| --- | --- | --- |
| **Characteristic** | **Differences** | **References** |
| LC_50_ for salt | Significantly higher LC_50_ in BW *Ae. aegypti* for the larva to adult transition which was an inheritable characteristic | Ramasamy et al., 2011;  Ramasamy et al., 2014;  Sivabalakrishnan et al., 2023 |
| Osmoregulatory  anal papillae in L3 larvae | Significantly larger anal papillae in JBW *Ae. aegypti* which was an inheritable characteristic | Surendran et al., 2018b |
| Haemolymph osmolarity in L4 larvae | (i) Increased concentration of amino acids in haemolymph for osmoconformation on limited exposure to BW of approximately 10 g/L salt  (ii) Haemolymph osmoconformation on 24 h exposure to BW of up to approximately 10 g/L salt | (i) Edwards, 1982  (ii) Kengne et al., 2019 |
| Voltage-gated ion channels in osmoregulatory epithelia of larval Malpighian tubules and anal papillae | Differential expression in larvae exposed to 10 g/L salt for 24 h | Farrel et al., 2024 |
| Aquaporins and ion transporters in L4 anal papillae | Differential expression in L4 exposed to 10.5 g/L salt for 24 h | Durrant et al., 2021 |
| Aquaporins in L4 anal papillae | Differential expression of aquaporins in L4 anal papillae epithelium in 7.5 g/L salt | Akhter et al., 2016 |
| Ion transport and ion transporters in the L4 gastric caecum | Differential localization and activities of Na^+^/K^+^ ATPase and V-type H^+^ ATPase in BW and FW reared L4 associated with differential ion fluxes | D’Silva et al., 2017 |
| Ion transport in the anal papillae and haemolymph ion concentrations in L4 | Decreased uptake of Na^+^ and Cl^-^ by anal papillae within 5h and increased haemolymph Na^+^, Cl^-^ and H^+^ concentrations within 6 h on transferring FW-adapted L4 to BW of approximately 10 g/L salt | Donini et al., 2007 |
| Gene expression  in mid-L4 larvae | Marked increases in transcripts for specific cuticle proteins (notably of the RR-2 family), signalling proteins, moulting hormone-related proteins, membrane transporters, enzymes involved in cuticle metabolism, enzymes concerned with synthesizing long chain hydrocarbons and cytochrome P450 monooxygenases in JBW L4. Also marked changes in long non-coding RNAs between JBW and JFW L4 | Ramasamy et al., 2021 |
| Protein composition of L4 cuticles | Marked differences between JBW and JFW compatible with gene expression data | Ramasamy et al., 2021 |
| Cuticle structure by TEM | Thicker endo-, exo- and whole procuticles with a tendency for more prominent lamellae and bouligands in JBW L4 larval bodies. Thinner procuticles in anal papillae of JBW L4 | Ramasamy et al., 2021 |
| Surfaces of shed L3 and L4 cuticles | More pronounced surface undulations in JBW *Ae. aegypti* cuticles by AFM and SEM | Sivabalakrishnan et al., 2023 |
| Egg sizes | Significantly smaller eggs in JBW *Ae. aegypti* | Sivabalakrishnan et al., 2023 |
| Surfaces of eggs by AFM and SEM | JBW *Ae.* *aegypti* egg surfaces were significantly less elastic by AFM, with more undulating surfaces seen by AFM and SEM | Sivabalakrishnan et al., 2023 |
| Hatchability of eggs and preimaginal development to adults | Hatchability of eggs and preimaginal development to adults of JFW *Ae. aegypti* is decreased in 10 gL^-1^ salt BW. These properties were maternally inherited in genetic crosses between JBW and JFW colony *Ae. aegypti* | Sivabalakrishnan et al., 2023 |
| Susceptibility of L3 and L4 to the common larvicide temephos | JBW *Ae. aegypti* were significantly more resistant in a 24h assay | Sivabalakrishnan et al., 2023 |

**Legend to Table S2.** BW: brackish water, FW: fresh water, JBW: Jaffna brackish water colony, JFW: Jaffna fresh water colony, L1-L4: first to fourth instar larval stages, AFM: atomic force microscopy, SEM: scanning electron microscopy, TEM: transmission electron microscopy, LC_50_: concentration producing 50% lethality.

**Table S3. Calculated mean and median values for thicknesses of exocuticles, endocuticles and whole procuticles for T1 tarsomere in different *Aedes aegypti* populations**

| **Mosquito population**  (number of mosquitoes sampled) | **Exocuticle**  Thickness in nm mean ± sd (median) | **Endocuticle**  Thickness in nm mean ± sd (median) | **Whole procuticle** Thickness in nm mean ± sd (median) |
| --- | --- | --- | --- |
| **JFW** G75 (n=9) | 1473 ± 238 (1452) | 984 ± 270 (958) | 2483 ± 486 (2397) |
| **JBW** G75 (n=9) | 1736 ± 227 (1722) | 1276 ± 333 (1272) | 3031 ± 527 (3010) |
| **JFWR** G10 (n=3) | 1597 ± 23 (1557) | 1002±250 (895) | 2638 ± 514 (2480) |
| **JBWR** G10 (n=3) | 1465± 237 (1401) | 1156 ± 34 (1159) | 2533± 560 (2388) |
| **BW field** (n=9) | 1724 ± 295 (1691) | 1245 ± 329 (1146) | 2953 ± 515 (3014) |

**Legend to Table S3.** BW: brackish water, JBW: Jaffna brackish water colony, JFW: Jaffna fresh water colony, JBWR: Jaffna brackish water reversal colony, JFWR: Jaffna fresh water reversal colony.

**Table S4. Calculated mean and median values for thicknesses of exocuticles, endocuticles and whole procuticles of abdomen in different *Aedes aegypti* populations**

**Legend to Table S4.** BW: brackish water, JBW: Jaffna brackish water colony, JFW: Jaffna fresh water colony, JBWR: Jaffna brackish water reversal colony, JFWR: Jaffna fresh water reversal colony.

| **Mosquito population**  (number of mosquitoes sampled) | **Exocuticle** Thickness in nm mean ± sd (median) | **Endocuticle** Thickness in nm mean ± sd (median) | **Whole procuticle** Thickness in nm mean ± sd (median) |
| --- | --- | --- | --- |
| **JFW** G75 (n=4) | 677 ± 90 (628) | 714 ± 212 (732) | 1372 ± 207 (1306) |
| **JBW** G75 (n=4) | 903 ± 182 (876) | 1124 ± 173 (1142) | 2058 ± 393 (1925) |
| **JFWR** G10 (n=4) | 775 ± 176 (730) | 711±99 (686) | 1526± 267 (1544) |
| **JBWR** G10 (n=4) | 821± 63 (831) | 553±29 (550) | 1316± 44 (1298) |
| **BW field** (n=6) | 1044 ± 166 (1059) | 902 ± 152 (857) | 1912 ± 290 (1902) |

**Table S5. Viable stages produced from eggs in the G1 to G7 generations when salinity was reversed in the JBWR and JFWR colonies**

| **Colony & Generation** | **Approximate starting number of eggs** | **Estimated percent viable stages resulting from eggs** | | | | | |
| --- | --- | --- | --- | --- | --- | --- | --- |
| **JBWR** |  | **L1** | **L2** | **L3** | **L4** | **Pupa** | **Adult** |
| G1 | 600 | 85 | 82 | 81 | 81 | 80 | 80 |
| G2 | 500 | 97 | 96 | 96 | 95 | 94 | 94 |
| G3 | 400 | 89 | 87 | 86 | 86 | 85 | 85 |
| G4 | 450 | 97 | 96 | 95 | 95 | 95 | 95 |
| G5 | 600 | 96 | 95 | 95 | 94 | 94 | 94 |
| G6 | 500 | 96 | 96 | 96 | 96 | 96 | 96 |
| G7 | 500 | 97 | 96 | 96 | 95 | 95 | 95 |
| **JFWR** |  | | | | | | |
| G1 | 600 | 35 | 16 | 15 | 12 | 11 | 09 |
| G2 | 450 | 43 | 20 | 17 | 14 | 13 | 11 |
| G3 | 600 | 46 | 19 | 17 | 15 | 15 | 14 |
| G4 | 500 | 71 | 58 | 56 | 55 | 55 | 54 |
| G5 | 600 | 76 | 72 | 70 | 70 | 70 | 70 |
| G6 | 550 | 77 | 75 | 74 | 74 | 74 | 74 |
| G7 | 500 | 90 | 89 | 89 | 89 | 89 | 89 |

**Legend to Table S5.** The approximate starting number of eggs used for hatching and the percent of different stages formed from the eggs are shown. JBWR: Jaffna brackish water reversal colony, JFWR: Jaffna fresh water reversal colony, L1-L4: first to fourth instar larval stages.

**Table S6. Reversibility of characteristics associated with preimaginal stages of Jaffna brackish water colony on transfer to fresh water**

| **Characteristic** | **Rate of reversal after transfer to fresh water** | **Reference** |
| --- | --- | --- |
| LC_50_ for salt in the L1 to adult transformation  which was an inheritable trait | Significant decrease in LC_50_ compared with JBW colony within five generations in FW | Ramasamy et al., 2014 |
| LC_50_ and LC_99_ of L3 and L4 for the larvicide temephos in a 24 h exposure assay | Significant decrease in LC_50_ and LC_99_ in 11 to 13 generations in FW to values not different from JFW and NFW colonies | Sivabalakrishnan et al., 2023 |

**Legend to Table S6.** FW: fresh water, JBW: Jaffna brackish water colony, JFW: Jaffna fresh water colony, NFW: Nawalapitiya fresh water colony, L1-L4: first to fourth instar larval stages, LC_50_: concentration producing 50% lethality, LC_99_: concentration producing 99% lethality.

**Additional references for supporting information**

Akhter, H., Misyura, L., Bui, P., Donini, A. (2017). Salinity responsive aquaporins in the anal papillae of the larval mosquito, Aedes aegypti. *Comparative Biochemistry and Physiology. Part A, Molecular & Integrative Physiology*, 203, 144–151. https://doi.org/10.1016/j.cbpa.2016.09.008

de Brito Arduino, M., Mucci, L. F., Serpa, L. L. N. (2010). Registro de larvas e pupas de *Aedes aegypti* e *Aedes albopictus* em recipientes com água salina em condições naturais. [Record of larvae and pupae of *Aedes aegypti* and *Aedes albopictus* in containers with saline water in natural conditions]. *Boletim Epidemiológico Paulista (Bepa)*, 7(83), 22-28

Donini, A., Gaidhu, M. P., Strasberg, D. R., & O'Donnell, M. J. (2007). Changing salinity induces alterations in haemolymph ion concentrations and Na+ and Cl- transport kinetics of the anal papillae in the larval mosquito, Aedes aegypti. *The Journal of Experimental Biology*, 210(Pt 6), 983–992. https://doi.org/10.1242/jeb.02732

Durant, A. C., Grieco Guardian, E., Kolosov, D., Donini, A. (2021). The transcriptome of anal papillae of *Aedes aegypti* reveals their importance in xenobiotic detoxification and adds significant knowledge on ion, water and ammonia transport mechanisms. *Journal of Insect Physiology*, 132, 104269. <https://doi.org/10.1016/j.jinsphys.2021.104269>

Edwards, H.A. (1982). Free amino acids as regulators of osmotic pressure in aquatic insect larvae. *Journal of Experimental Biology*, 101, 153–160. https://doi.org/10.1242/jeb.101.1.153

Farrell, S., Dates, J., Ramirez, N., Hausknecht-Buss, H., Kolosov, D. (2024). Voltage-gated ion channels are expressed in the Malpighian tubules and anal papillae of the yellow fever mosquito (*Aedes aegypti*), and may regulate ion transport during salt and water imbalance. *The Journal of Experimental Biology*, 227(3), jeb246486. https://doi.org/10.1242/jeb.246486

Idris, F. H., Usman, A., Surendran, S. N., Ramasamy, R. (2013). Detection of Aedes albopictus pre-imaginal stages in brackish water habitats in Brunei Darussalam. *Journal of Vector Ecology*, 38(1), 197–199. <https://doi.org/10.1111/j.1948-7134.2013.12029.x>

Kengne, P., Charmantier, G., Blondeau-Bidet, E., Costantini, C., Ayala, D. (2019). Tolerance of disease-vector mosquitoes to brackish water and their osmoregulatory ability. *Ecosphere*, 10, e02783. <https://doi.org/10.1002/ecs2.2783>

Ratnasari, A., Jabal, A.R., Rahma, N., Rahmi, S., Karmila, M., Wahid, I. (2020). The ecology of *Aedes aegypti* and *Aedes albopictus* larvae habitat in coastal areas of South Sulawesi, Indonesia. *Biodiversitas* 21, 4648-4654. https://doi.org/10.13057/biodiv/d211025.

Shamna, A.K., Vipinya, C., Sumodan, P.K. (2022). Detection of *Aedes albopictus* (Diptera: Culicidae) breeding in brackish water habitats in coastal Kerala, India and its implications for dengue scenario in the state. *International Journal of Entomology Research*, 7, 214–216.

D'Silva, N. M., Patrick, M. L., O'Donnell, M. J. (2017). Effects of rearing salinity on expression and function of ion-motive ATPases and ion transport across the gastric caecum of *Aedes aegypti* larvae. *The Journal of Experimental Biology*, 220(Pt 17), 3172–3180. https://doi.org/10.1242/jeb.163170

Surendran, S.N., Sivabalakrishnan, K., Jayadas, T.T.P., Santhirasegaram, S., Laheetharan, A., Senthilnanthanan, M., et al. (2018b). Adaptation of *Aedes aegypti* to salinity: Characterized by larger anal papillae in larvae. *Journal of Vector Borne Diseases,* 55, 235-238.
